# Sustained androgen receptor signaling is a determinant of melanoma cell growth potential and tumorigenesis

**DOI:** 10.1084/jem.20201137

**Published:** 2020-10-28

**Authors:** Min Ma, Soumitra Ghosh, Daniele Tavernari, Atul Katarkar, Andrea Clocchiatti, Luigi Mazzeo, Anastasia Samarkina, Justine Epiney, Yi-Ru Yu, Ping-Chih Ho, Mitchell P. Levesque, Berna C. Özdemir, Giovanni Ciriello, Reinhard Dummer, G. Paolo Dotto

**Affiliations:** 1 Department of Biochemistry, University of Lausanne, Epalinges, Switzerland; 2 Department of Computational Biology, University of Lausanne, Lausanne, Switzerland; 3 Swiss Institute of Bioinformatics, Lausanne, Switzerland; 4 Cutaneous Biology Research Center, Massachusetts General Hospital, Charlestown, MA; 5 Department of Dermatology, Harvard Medical School, Boston, MA; 6 Department of Oncology, University of Lausanne, Ludwig Institute for Cancer Research Lausanne, Epalinges, Switzerland; 7 Department of Dermatology, University Hospital Zürich, University of Zürich, Zürich, Switzerland; 8 Department of Oncology, Centre Hospitalier Universitaire Vaudois, Lausanne, Switzerland; 9 International Cancer Prevention Institute, Epalinges, Switzerland

## Abstract

Melanoma susceptibility differs significantly in male versus female populations. Low levels of androgen receptor (AR) in melanocytes of the two sexes are accompanied by heterogeneous expression at various stages of the disease. Irrespective of expression levels, genetic and pharmacological suppression of AR activity in melanoma cells blunts proliferation and induces senescence, while increased AR expression or activation exert opposite effects. *AR* down-modulation elicits a shared gene expression signature associated with better patient survival, related to interferon and cytokine signaling and DNA damage/repair. AR loss leads to dsDNA breakage, cytoplasmic leakage, and STING activation, with AR anchoring the DNA repair proteins Ku70/Ku80 to RNA Pol II and preventing RNA Pol II–associated DNA damage. AR down-modulation or pharmacological inhibition suppresses melanomagenesis, with increased intratumoral infiltration of macrophages and, in an immune-competent mouse model, cytotoxic T cells. AR provides an attractive target for improved management of melanoma independent of patient sex.

## Teaser

Ma et al. uncovers an essential role of AR signaling in melanoma cell expansion and tumorigenesis, with loss of AR activity inducing cellular senescence, genomic DNA breakage, a STING-dependent inflammatory cascade, and immune cell recruitment, providing an attractive venue for new combination approaches to disease management.

## Graphical Abstract

**Figure:**
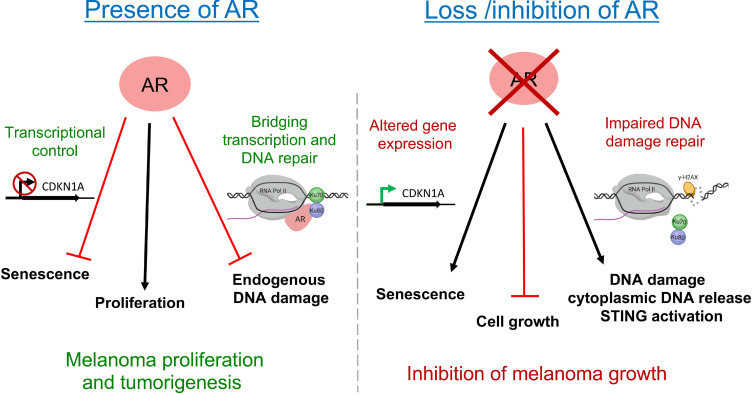

## Introduction

Malignant melanoma is the fifth most common cancer in the world, and its incidence is rising. Among the many prognostic risk factors that have been proposed for the disease, one of the most intriguing and least understood is sex (Nosrati and Wei, 2014). In fact, melanoma is an example of primary clinical significance for investigating sex-related differences in cancer incidence and survival, with the male population having greater susceptibility than the female, across all ages (Nosrati and Wei, 2014). Although differences in lifestyle and behavior may explain the delay and higher disease stage in men at diagnosis, the female survival advantage persists even after adjusting for these and additional variables (histological subtypes, Breslow thickness, and body site; Gamba et al., 2013; Joosse et al., 2013).

As for sexual dimorphism in other cancer types (Clocchiatti et al., 2016), even for susceptibility to melanoma, differences in sex hormone levels and/or downstream pathways are likely to play a key role (Nosrati and Wei, 2014). Relative to sex protein hormones, much more evidence exists on the impact of sex steroid hormones on cancer development (Clocchiatti et al., 2016). The great majority of accrued information for melanoma relates to estrogen signaling, while much less is known about androgen signaling.

In experimental settings, estrogen signaling was found to restrict melanocyte proliferation, enhance differentiation, and suppress melanoma development (Natale et al., 2016, 2018; Ramelyte et al., 2017). In spite of the experimental evidence, epidemiological studies on the interconnection between estrogen levels and melanoma development and progression yield conflicting conclusions (Nosrati and Wei, 2014; Ramelyte et al., 2017), which may be due, in part, to the difficulty in controlling for estrogen levels, which vary with the menstrual cycle, onset of menopause, use of oral contraceptives, and hormone replacement therapy. Additionally, the possible interplay between estrogens and other hormones, specifically androgens, has not been taken into consideration. An interplay with frequently opposite effects between estrogen and androgen signaling has been reported for several cell types (Clocchiatti et al., 2016), which may extend to melanocytes.

The androgen receptor (AR) is expressed in many cell types and, while most studies have focused on prostate cancer, AR signaling has been implicated in tumorigenesis in other organs, specifically breast, bladder, kidney, lung, and liver (Chang et al., 2014). Surprisingly little is known about the role of AR signaling in melanoma. As early as 1980, it was proposed that differences in androgen levels could explain the lower survival of male melanoma patients than females (Rampen and Mulder, 1980). Since then, however, only circumstantial pharmacological evidence has been obtained, pointing to a positive role of AR signaling in development of the disease (Nosrati and Wei, 2014). For instance, in a human melanoma cell line expressing an atypical form of AR, incubation with androgens significantly stimulated proliferation, with effects that were reversed by treatment with the androgen antagonist flutamide (or its active metabolite hydroxyflutamide; Morvillo et al., 1995). The nonsteroidal antiandrogen flutamide was also found to be effective in diminishing tumor growth and increasing survival of nude mice inoculated with human melanoma cells through possibly indirect effects (Morvillo et al., 2002). In fact, others reported that administration of flutamide increased murine splenocyte proliferation and IFN secretion in response to irradiated murine B16 melanoma cells, and when flutamide was administered with an irradiated B16 vaccine, this combination improved the survival of mice implanted with nonirradiated B16 cells (Hsueh et al., 2003). Despite the above, genetic evidence in support of an intrinsic role of AR signaling in melanoma development is missing, with the possible exception of a study of a melanoma cell line with or without infection with a single shRNA silencing vector, which resulted in limited AR down-modulation (Wang et al., 2017). AR signaling in this setting was implicated in the control of melanoma cells’ invasive properties, without any effect on proliferation.

In this study, based on analysis of a large panel of clinical samples and melanoma cells from both male and female patients, we show that, irrespective of expression levels, genetic and pharmacological suppression of AR activity triggers melanoma cell senescence and limits tumorigenesis, eliciting a gene expression signature related to IFN and inflammatory cytokines and associated with better patients’ survival. Loss of AR activity in both melanoma cells and tumors is sufficient to cause massive chromosomal DNA breakage and leakage into the cytoplasm, with a stimulator of IFN genes (STING)–dependent inflammatory signaling cascade. Underlying these events, we find that AR is essential in melanoma cells for anchoring the DNA repair proteins Ku70/Ku80 to RNA polymerase II (Pol II) and preventing RNA Pol II–associated DNA damage. Although at different levels, androgens are produced in both male and female individuals, and AR targeting provides an attractive therapy approach for improved management of melanoma irrespective of patient sex.

## Results

### AR is heterogeneously expressed in melanocytic lesions and melanoma cells

**Figure 1.**
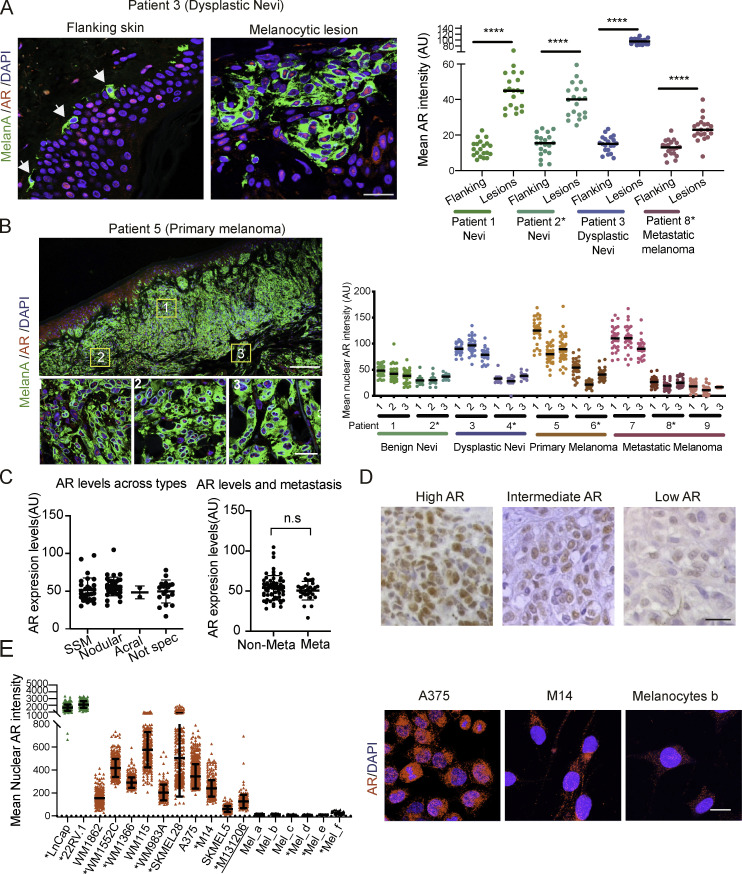
**AR expression in melanoma cells. (A)** Representative images and quantification of AR expression in cells of melanocytic lesions versus melanocytes of flanking normal skin (stars) by double IF with antibodies against AR (red) and MelanA (green) for melanocyte identification. DAPI was used for nuclear localization (blue). Shown are AR fluorescence signal intensity in arbitrary units (AU) per individual cells together with mean and statistical significance. MelanA-positive cells, *n* ≥ 25, unpaired *t* test, ****, P < 0.001. Samples from male patients in this and following panels are indicated by asterisks. **(B)** Left: Double IF staining of a primary melanoma lesion and topographically distinct areas (boxes 1, 2, and 3) analyzed for single-cell AR expression. Scale bars: 500 and 50 µm, respectively. IF images of cells in this and other lesions are shown in Fig. S2. Right: Quantification of nuclear AR fluorescence signal in individual MelanA-positive cells (dots) from three topographically delimited areas per melanocytic lesion of different patients. Fluorescence intensity AU values per individual cells are indicated together with the mean. MelanA-positive cells, *n* ≥ 50, unpaired *t* test, ****, P < 0.001. **(C)** Quantification of AR fluorescence signal in MelanA-positive cells in a tissue microarray of different melanoma lesions (left) and metastatic and nonmetastatic forms (right). SSM, superficial spreading melanoma; acral, acral lentiginous melanoma. Quantification was based on digitally acquired images of three independent fields per lesion (≥50 cells per field), with averaged values per individual lesion shown together with mean. Quantification of samples divided by sex and age of patients is provided in Fig. S1 E. Patient sample details provided in Table S1. n.s, not significant. **(D)** Immunohistochemical staining with anti-AR antibodies of melanomas with high versus intermediate and low AR expression as assessed by double IF analysis in A. Scale bar: 30 µm. Lower-magnification images with MelanA staining of parallel sections are shown in Fig. S1 F. **(E)** Quantification and representative images of nuclear AR expression by IF analysis of the indicated melanoma cell lines or primary melanoma cells versus primary melanocytes (Mel a-f), and prostate cancer cell lines (LnCAp, 22RV.1) examining >100 cells per sample. Shown are individual cells values (dots) together with mean ± SD. Scale bar: 10 µm. Additional images of cells are shown in Fig. S1 G.

**Figure S1.**
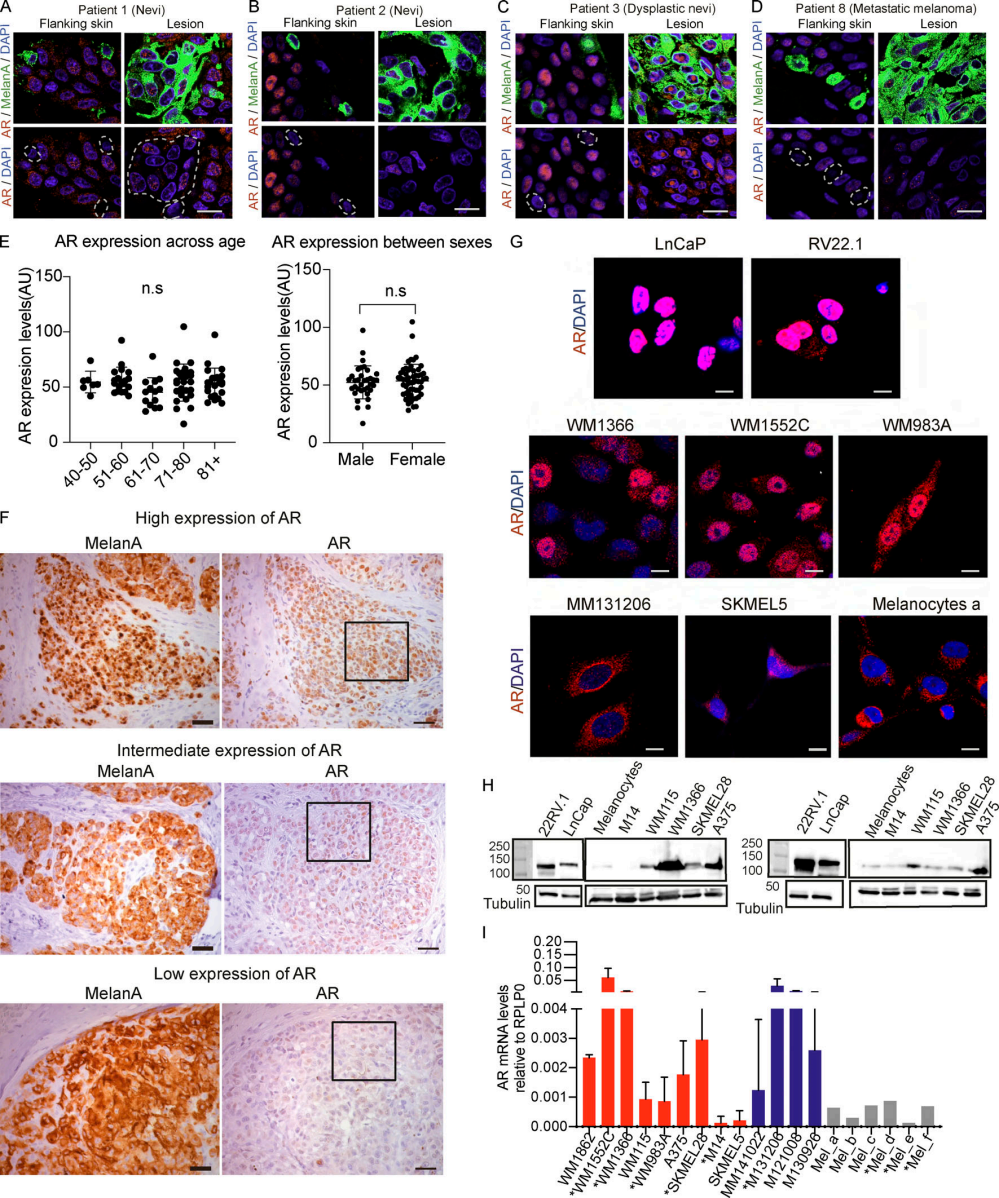
**AR expression analysis of patient-derived melanocytic lesions and melanoma cells. (A–D)** Double IF images of benign nevi (A and B), dysplastic nevi (C), and metastatic melanoma (D) in parallel with flanking skin stained with anti-MelanA (green) and anti-AR (ab74272; red) antibodies. Highlighted in the lower panels are representative MelanA-positive cells and areas used for quantification in Fig. 1 A. Scale bar: 10 µm. **(E)** Quantification of AR fluorescence signal in MelanA-positive cells in a tissue microarray of melanoma patients divided by age or sex. Quantification was based on digitally acquired images of three independent fields per clinical lesion (minimum of 50 cells per field) on the arrays. Results are expressed as average values for each lesion (dots) together with mean across years of age (left) or sex (right) of patients. n.s, not significant. **(F)** Immunohistochemical staining with anti-MelanA and anti-AR (ab74272) antibodies of parallel sections of different melanomas with high, intermediate, and low levels of AR expression as quantified by double IF analysis in Fig. 1 B. Shown are representative images, with only the enlarged boxed areas shown in Fig. 1 D. Scale bar: 50 µm. **(G)** Representative IF images of the indicated prostate cancer cells lines (LnCaP and 22RV.1), melanoma cell lines and primary melanoma cells with high (WM1366, WM1552C, and WM983A) and low (MM131206 and SKMEL5) AR expression, and primary human melanocytes (strain a) stained with anti-AR (red) antibody (D6F11) and DAPI (blue) nuclear staining. Scale bar: 10 µm. **(H)** Immunoblot analysis of AR expression in melanoma cell lines (A375, SKMEL28, WM1366, WM115, and M14) and primary human melanocytes with two different antibodies in parallel with prostate cancer cell lines (LnCaP and 22RV.1) as comparison. All extracts were run in two parallel gels and blotted, respectively, with anti-AR (D6F11; left) or anti-AR (PG-21; right) antibodies. Shown are low- and high-exposure images of the same blots, for better AR detection in highly expressing prostate cancer versus melanoma cells. **(I)** RT-qPCR analysis of *AR* mRNA expression in a panel of melanoma cell lines (red), early-passage primary melanoma cells (blue), and primary human melanocytes (gray). Results are expressed as relative to RRLP0 values.

**Figure S2.**
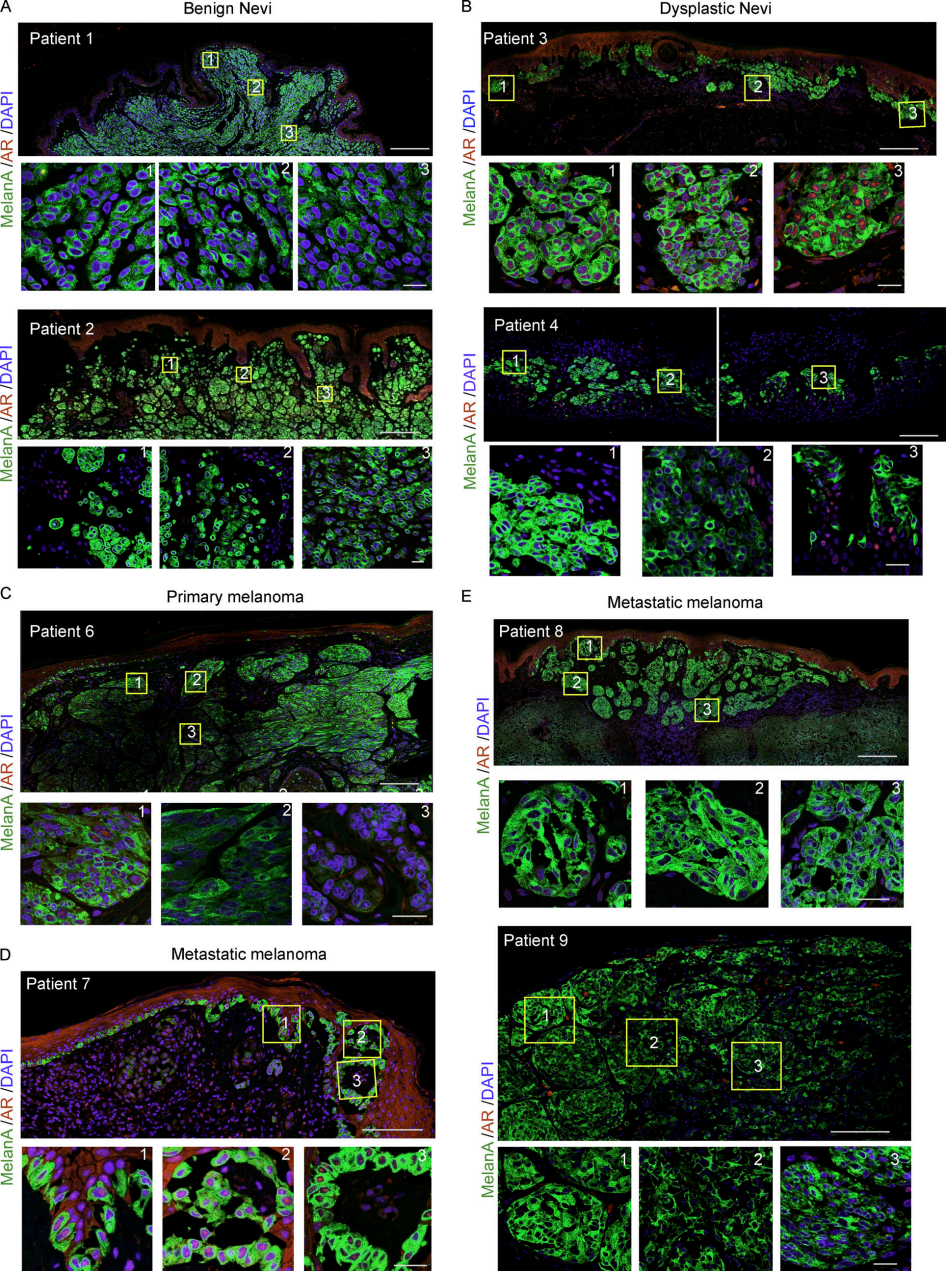
**Double IF analysis of patient-derived melanocytic lesions. (A–D)** IF staining of benign nevi (A, patients 1 and 2), dysplastic nevi (B, patients 3 and 4), primary melanoma (C, patient 6), and metastatic melanoma (D, patients 7, 8, and 9) skin tissues with anti-MelanA (green) and anti-AR (ab74272; red) antibodies, and topographically distinct areas (boxes 1, 2, and 3) used for single-cell AR expression quantification in Fig. 1 B. Shown are representative low- and high-magnification images of the areas used for quantification. Scale bar: 2 mm and 20 µm, respectively.

Melanoma tumors are characterized by distinct phenotypic states and display significant intra- and intertumor heterogeneity (Tirosh et al., 2016). Double immunofluorescence (IF) analysis of melanocytes in benign or dysplastic nevi or metastatic melanoma versus melanocytes from flanking skin showed consistently increased levels of AR expression in the melanocytic lesions, with heterogeneity of AR protein expression at the single-cell level (Fig. 1 A and Fig. S1, A–D). Analysis of multiple topographically distinct areas within multiple lesions showed no significant intralesional heterogeneity in AR expression, while confirming variations among lesions (Fig. 1 B and Fig. S2). Similar IF analysis of melanoma tissue microarrays also showed variable degrees of AR expression irrespective of stages of neoplastic development or sex and age of patients (Fig. 1 C, Fig. S1 E, and Table S1). Immunohistochemical staining confirmed the IF results with prevalent nuclear AR localization in lesions with elevated and intermediate expression and more uneven localization when expressed at low levels (Fig. 1 D and Fig. S1 F).

Immunostaining of cultured cells also showed a variation in AR protein expression among various melanoma cell lines and primary melanoma cells derived from male or female patients, with AR levels being uniformly low in primary melanocytes (Fig. 1 E, Fig. S1 G, and Table S2). As observed in vivo, AR localization was largely nuclear in melanoma cells with elevated expression, similar to LnCAP or 22RV.1 prostate cancer cell lines, while in melanoma cell lines or primary melanocytes with low AR levels, there was limited punctate nuclear localization with prevalent perinuclear distribution (Fig. 1 E and Fig. S1 G).

Variations in AR expression were further confirmed by immunoblotting with two different antibodies, with a similar pattern of bands, and by quantitative RT-PCR (RT-qPCR) analysis of melanoma cell lines, early passage primary melanoma cells, and primary melanocytes, which were again found to express lower AR levels (Fig. S1, H and I).

### Sustained AR expression is required for melanoma cell proliferation and self-renewal potential

**Figure S3.**
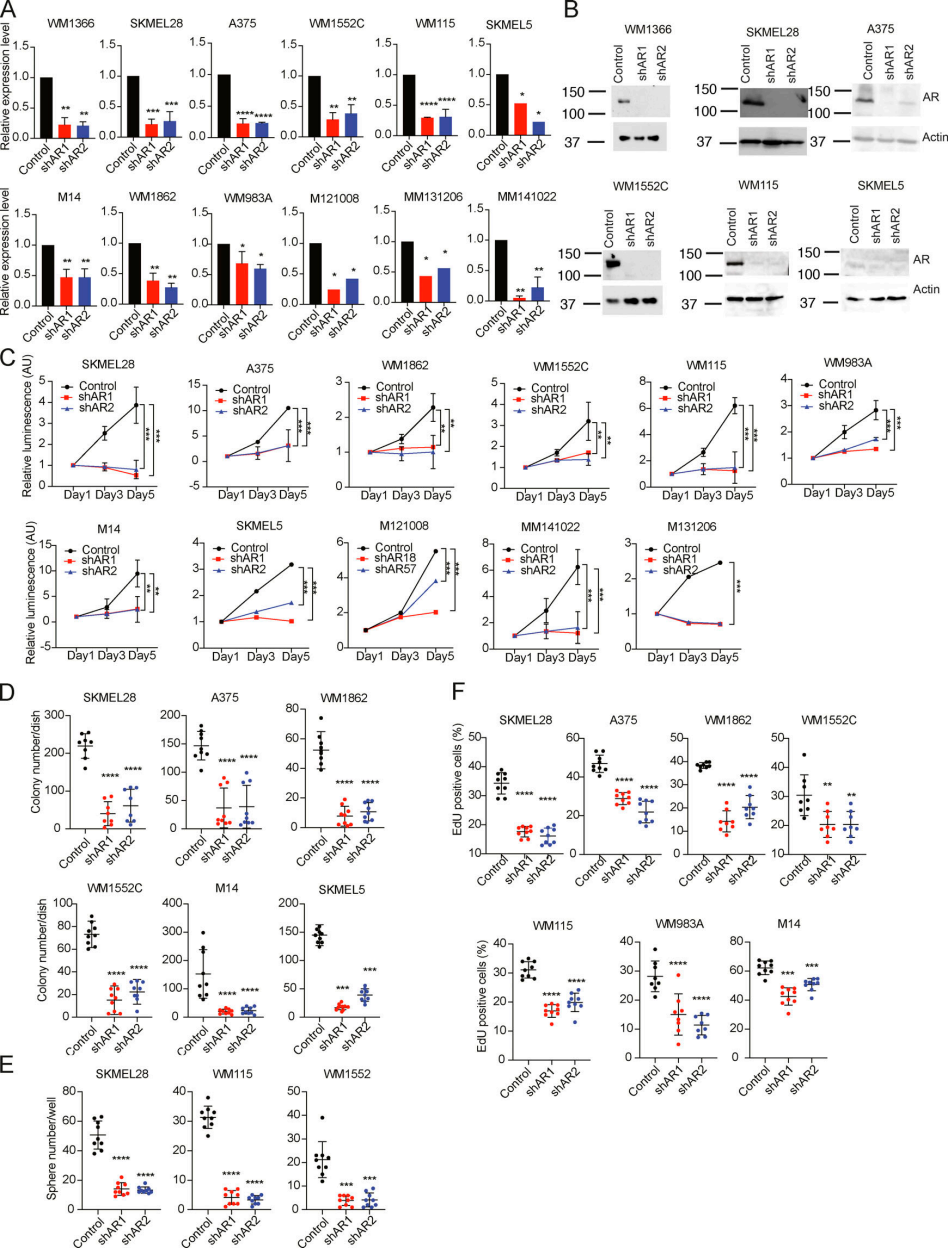
**AR down-modulation suppresses melanoma cell growth. (A)** Down-modulation of *AR* expression in a panel of melanoma cell lines and primary melanoma cells (M121008, MM131206, and MM141022) infected with two *AR*-silencing lentiviruses versus empty control as assessed by RT-qPCR after selection. Data are shown as mean ± SD, one-way ANOVA with Dunnett’s test, *, P < 0.05; **, P < 0.01; ***, P < 0.005; ****, P < 0.001. Cultures, *n* = 6. **(B)** Immunoblot analysis of AR protein expression in different melanoma cell lines with or without *AR* gene silencing as in A. Shown are the immunoblots of AR protein levels after densitometric scanning of the autoradiographs, using actin signal for normalization (lower panels). **(C)** Cell density assays (CellTiter-Glo) were performed with the indicated melanoma cell lines and primary melanoma cells (M121008, MM131206, and MM141022) infected with two *AR*-silencing lentiviruses versus empty vector control. Results are presented as luminescence intensity values relative to day 1. Data are shown as mean ± SD, one-way ANOVA with Dunnett’s test, **, P < 0.01; ***, P < 0.005. Cultures, *n* = 9. **(D–F)** Colony formation, sphere formation, and EdU incorporation assays with indicated melanoma cell lines with or without *AR* silencing. Shown are the results of three independent experiments quantifying in each case three culture dishes per condition (indicated by dots, mean ± SD). Results are presented as mean ± SD, one-way ANOVA with Dunnett’s test, **, P < 0.01; ***, P < 0.005; ****, P < 0.001. Cultures, *n* = 9.

**Figure 2.**
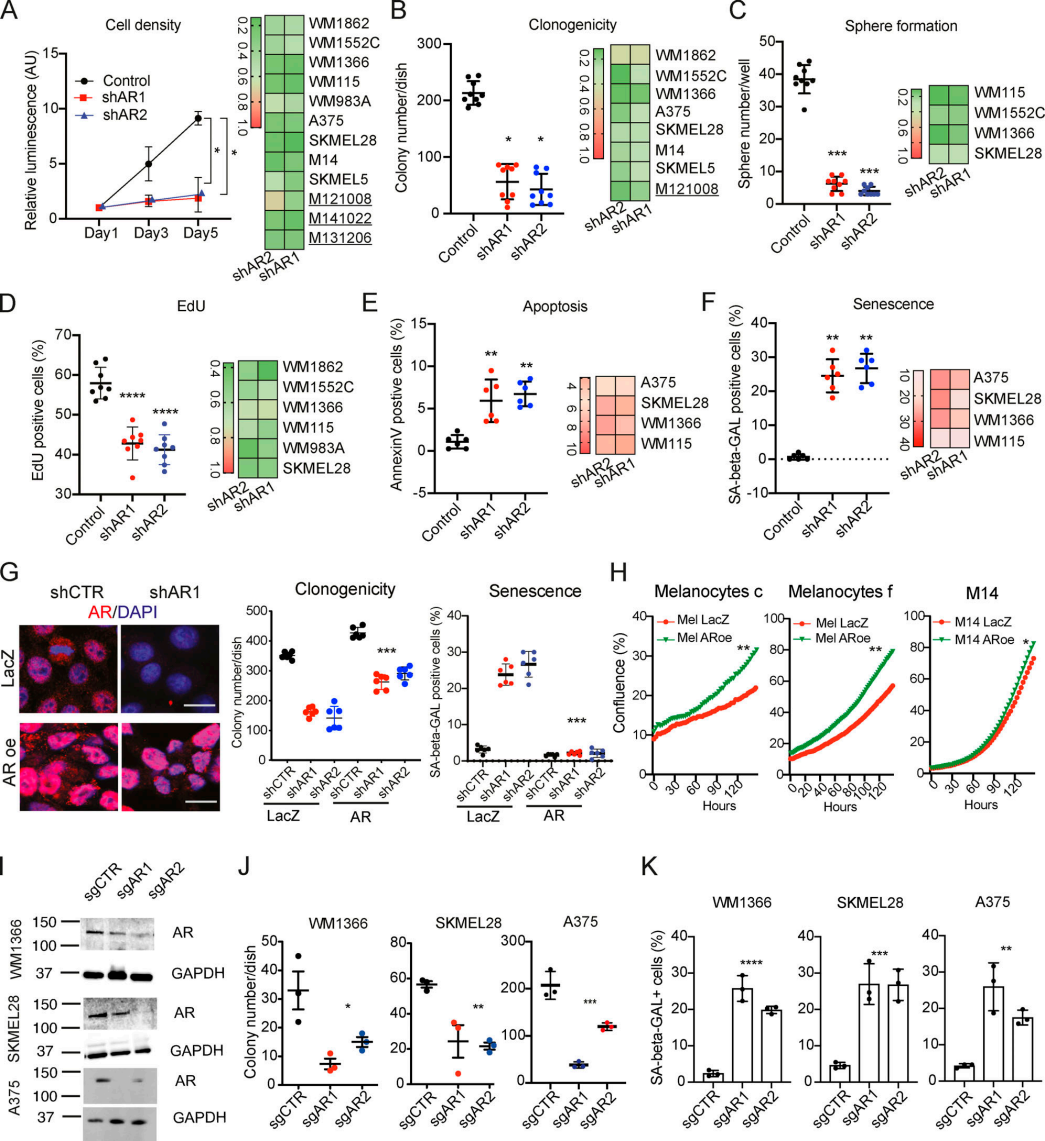
**AR down-modulation suppresses melanoma cell proliferation potential. (A)** Left: WM1366 melanoma cells infected with two *AR*-silencing lentiviruses versus empty vector control were analyzed by cell density assays (CellTiter-Glo) on the indicated days after selection. Shown are luminescence intensity values relative to day 1 ± SD; one-way ANOVA with Dunnett’s test. Cultures, *n* = 9; all experiments repeated three times. *, P < 0.05. Right: Heatmap results with additional melanoma cells. Efficiency of *AR* gene silencing and individual plots for all heatmap results are shown in Fig. S3 (A and B). **(B and C)** Clonogenicity and sphere formation assays of the same WM1366 together with heatmap results with additional melanoma cells. Results are shown as individual cultured dishes together with mean ± SD; one-way ANOVA with Dunnett’s test. Cultures, *n* = 9; all experiments repeated three times. *, P < 0.05; ***, P < 0.005. **(D–F)** Melanoma cells with or without *AR* silencing as in the previous panels were tested by EdU labeling assay (D), annexin V staining (E), or SA-β gal activity (F). Shown are individual plots for WM1366 melanoma cells together with mean ± SD; one-way ANOVA with Dunnett’s test; heatmap results for all other indicated lines. Cultures, *n* = 6; all experiments repeated two times. **, P < 0.01; ****, P < 0.001. **(G)** Right: IF analysis of AR expression in A375 cells stably infected with an AR-overexpressing (AR oe) lentivirus or vector control and superinfected with an *AR*-silencing lentivirus or corresponding control. Scale bar 10 µm. Quantification of results, also in cells infected with a second *AR-*silencing lentivirus, together with mRNA expression measurements are shown in Fig. S4 (C and D). Left: Clonogenicity and SA-β gal assays of A375 melanoma cells with or without *AR* silencing and overexpression as indicated. Data are shown as mean ± SD; one-way ANOVA with Dunnett’s test. Cultures, *n* = 6; all experiments repeated two times. ***, P < 0.005. Cell density, EdU labeling, and apoptosis assays for the same cells are shown in Fig. S4 E. **(H)** Proliferation live-cell imaging assays (IncuCyte) of the indicated primary melanocyte strains (c and f) and melanoma cells (M14) stably infected with an AR-overexpressing lentivirus versus empty vector control. Cells were plated in triplicate wells in 96-well plates followed by cell density measurements (four images per well every 4 h for 128 h). Cultures, *n* = 3; Pearson r correlation test. *, P < 0.05; **, P < 0.01. **(I)** Immunoblot analysis of AR expression in dCas9-KRAB–expressing melanoma cells (WM1366, SKMEL28, and A375) infected with lentiviruses expressing two sgRNAs targeting the *AR* promoter region (sgAR1 and sgAR2) versus scrambled sgRNA control (sgCTR) for 3 d. **(J and K)** Parallel cultures of cells as in I were tested by clonogenicity (J) and SA-β gal (K) assays on triplicate dishes, starting on day 3 after sgRNA expression. Cultures, *n* = 3 biological replicates; one-way ANOVA with Dunnett’s test. *, P < 0.05; **, P < 0.01; ***, P < 0.005; ****, P < 0.001.

**Figure S4.**
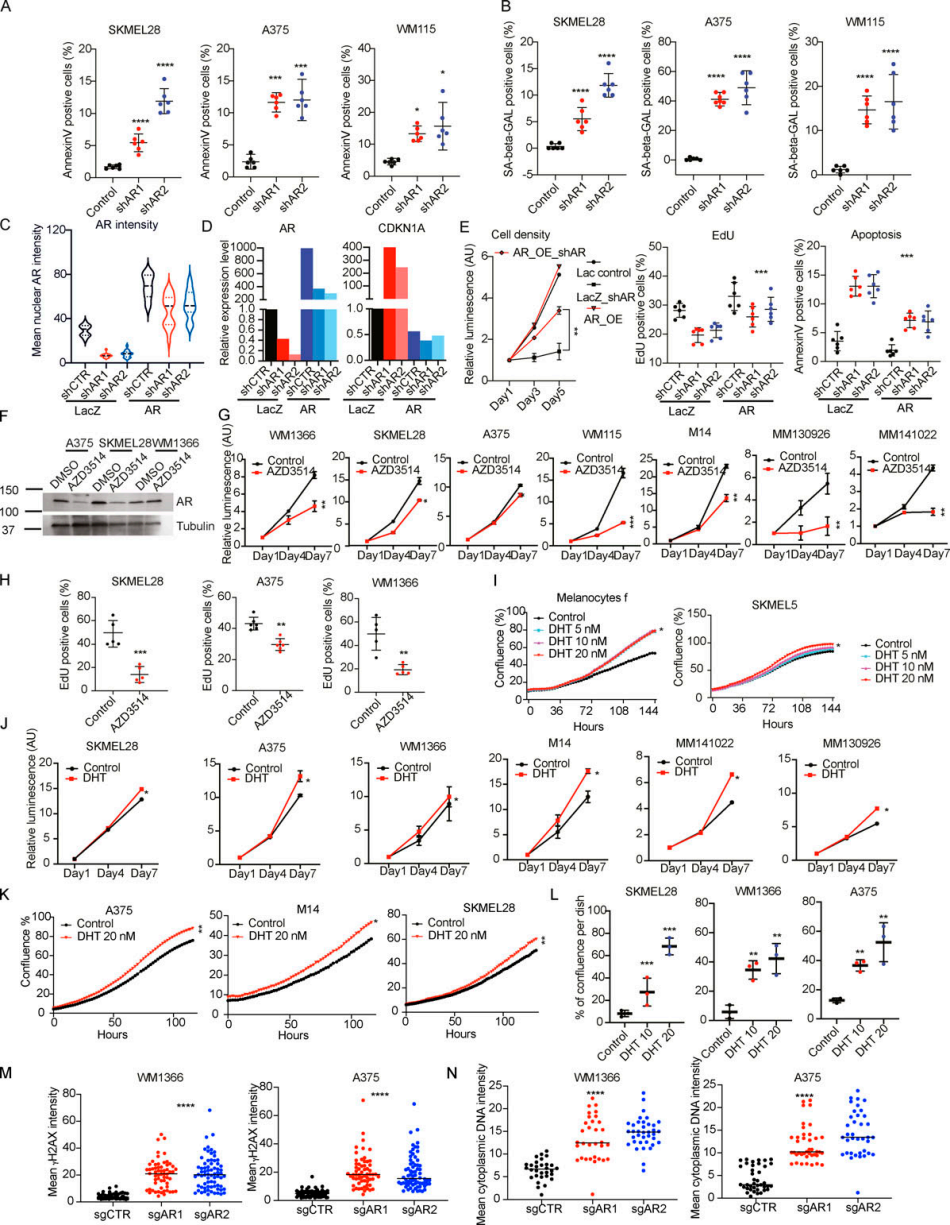
**Rescue of *AR*-silencing effects, pharmacological inhibition, and agonist stimulation. (A and B)** Apoptosis and senescence assays in melanoma cells with or without *AR* silencing. Indicated melanoma cell lines infected with two *AR*-silencing lentiviruses versus empty vector control were tested by AnnexinV staining (A) and SA-β-GAL staining (B) after selection. AnnexinV- and SA-β-GAL–positive cells were counted using ImageJ. Shown are representative images and results of two independent experiments quantifying in each case three culture dishes per condition (indicated by dots, mean ± SD), one-way ANOVA with Dunnett’s test, *, P < 0.05; **, P < 0.01; ***, P < 0.005, ****, P < 0.001. Cultures, *n* = 6. **(C–E)** Concomitant AR overexpression suppresses *AR*-silencing effects. A375 cells stably infected with a lentiviral vector for constitutive AR expression versus LacZ control and superinfected with two *AR*-silencing lentiviruses versus vector control for 5 d. Shown is a violin plot quantifying AR IF signal intensity (C), with corresponding representative images shown in Fig. 2 G. Cells per condition, *n* > 20, one-way ANOVA with Dunnett’s test, ****, P < 0.001. **(D)** Quantification of *AR* mRNA expression by RT-qPCR analysis of A375 cells with or without AR overexpression and silencing as in C. The same samples were analyzed for levels of *CDKN1A* expression as a marker/effector of cellular senescence induced by *AR* gene silencing. **(E)** The same melanoma cells as in C and D were tested by cell density assays (CellTiter-Glo), EdU incorporation assays, or apoptosis by annexin V staining. For each condition, cells were tested in duplicated culture dishes, with all experiments repeated three times. Data are shown as mean ± SD, one-way ANOVA with Dunnett’s test, **, P < 0.01; ***, P < 0.005. Cultures, *n* = 6. **(F and G)** Growth-suppressive effects of AR inhibitors on melanoma cells. (F) Immunoblot analysis of AR protein expression in the indicated melanoma cell lines treated with AZD3514 (10 µM for 48 h) versus DMSO control. **(G)** Cell density assays (CellTiter-Glo) of the indicated melanoma cell lines and primary melanoma cells (MM130926 and MM141022) treated with AZD3514 (10 µM) versus solvent control (DMSO). Cells were plated on triplicate wells in 96-well dishes followed by cell density/metabolic activity measurements on the indicated days after treatment. Results are presented as luminescence intensity values relative to day 1. Data are shown as mean ± SD, *, P < 0.05; **, P < 0.01. *t* test. Cultures, *n* = 6. **(H)** EdU labeling assays of the indicated melanoma cells treated with AZD3514 (10 µM) versus solvent control (DMSO) on day 5 after treatment. Data are shown as mean ± SD, *t* test, **, P < 0.01; ***, P < 0.005. Cultures, *n* = 5. **(I and J)** Growth-stimulatory effects of DHT treatment of melanoma cells. **(I)** Proliferation live-cell imaging assays (IncuCyte) of the primary melanocytes (strain f) and SKMEL5 melanoma cells treated with different doses of DHT (5, 10, and 20 nM) versus DMSO control. Cultures, *n* = 3; Pearson r correlation test, *, P < 0.05. **(J)** Cell density assays (CellTiter-Glo) of the indicated melanoma cell lines and primary melanoma cells (MM130926 and MM141022) treated with the AR agonist DHT (20 nM) versus solvent control (DMSO) on the indicated days after treatment. Results are presented as luminescence intensity values relative to day 1. **(K)** Proliferation live-cell imaging assays of the indicated melanoma cells treated with DHT (20 nM) versus DMSO control. Cultures, *n* = 3; Pearson r correlation test, *, P < 0.05; **, P < 0.01. **(L)** Cell density assays of the indicated melanoma cells tested under very sparse conditions. Cells were cultured in medium with charcoal-treated serum for 48 h followed by plating at very low numbers (500 cells per 60-mm dish) in the same medium ± treatment with DHT (10 and 20 nM) versus solvent control (DMSO) for 7 d. Data are represented as relative cell density as quantified by ImageJ analysis of crystal violet–stained dishes. one-way ANOVA with Dunnett’s test, *, P < 0.05; **, P < 0.01; ***, P < 0.005. Cultures, *n* = 3. **(M and N)** Quantification of nuclear γ-H2A and cytoplasmic DNA IF signal intensity in the indicated melanoma cells with or without CRISPRi-mediated downmodulation of AR expression as shown in Fig. 2 I. More than 100 cells were counted in each condition. Results are expressed as mean. Cultures, *n* = 3; one-way ANOVA with Dunnett’s test, ****, P < 0.001.

The heterogeneous levels of AR expression raised the question of its biological significance. Accordingly, we silenced *AR* expression in a panel of melanoma cells harboring either *BRAF* or *NRAS* mutations individually and in combination with *TP53, PTEN*, and/or *CDK4* mutations (Fig. S3, A and B). Irrespective of basal levels of AR expression, in all cases silencing of the gene by two different shRNAs resulted in drastically reduced proliferation and self-renewal as assessed by cell density, clonogenicity, and sphere formation assays (Fig. 2, A–C; and Fig. S3, C–E). Effects were paralleled by decreased DNA synthesis, induction of apoptosis, and cellular senescence (Fig. 2, D–F; Fig. S3 F; and Fig. S4, A and B). The shRNA gene silencing effects were suppressed in melanoma cells concomitantly infected with an AR-overexpressing lentivirus (Fig. 2 G and Fig. S4, C–E), which was by itself sufficient to enhance proliferation of primary melanocytes as well as melanoma cells with low AR expression (Fig. 2 H).

As an alternative to shRNA-mediated gene silencing, we also down-modulated AR expression by a CRISPR interference (CRISPRi) system (Ho et al., 2017; Kearns et al., 2014), whereby a dCas9-KRAB transcription repressor was directed to the *AR* promoter region by two different single-guide RNAs (sgRNAs). Mass infection of dCas9-KRAB–expressing melanoma cells with two lentiviruses with *AR*-targeting sgRNAs significantly reduced AR protein levels, decreased clonogenicity, and induced cellular senescence, reproducing the effects of *AR* gene silencing (Fig. 2, I–K).

### Modulation of melanoma and melanocyte proliferation by pharmacological inhibition and agonist stimulation

**Figure 3.**
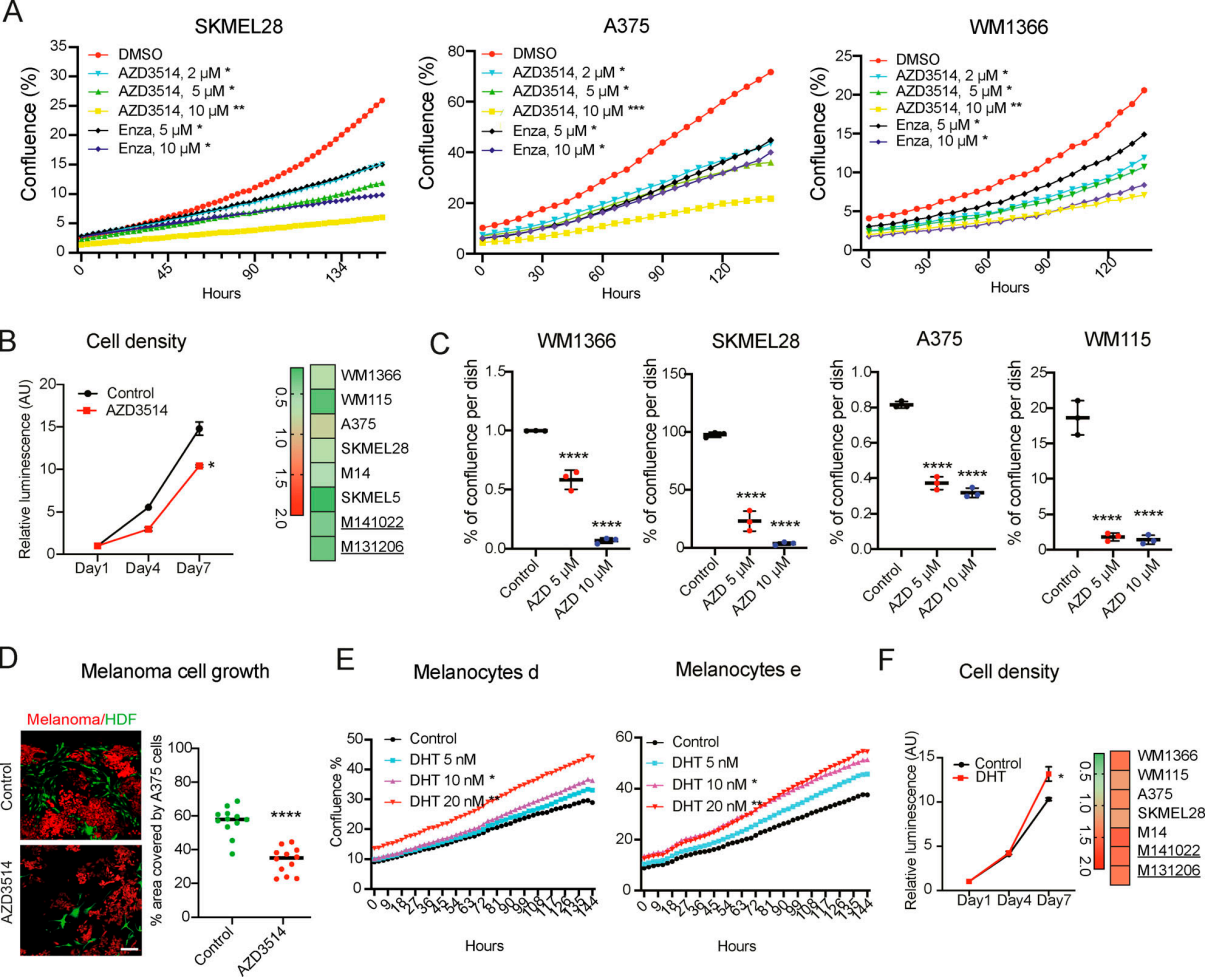
**Modulation of melanoma cell proliferation by pharmacological inhibition and agonist stimulation. (A)** Proliferation live-cell imaging assays (IncuCyte) of the indicated melanoma cell lines treated with the AR inhibitors AZD3514 (2, 5, and 10 µM) or Enzalutamide (5 and 10 µM) versus DMSO control. Number of wells, *n* = 3; Pearson r correlation test. *, P < 0.05; **, P < 0.01; ***, P < 0.005. **(B)** WM1366 melanoma cells treated with AZD3514 versus DMSO control were analyzed by cell density assays (CellTiter-Glo) on the indicated days. Data are shown as mean ± SD; one-way ANOVA with Dunnett’s test. Cultures, *n* = 9; all experiments repeated three times. *, P < 0.05. Right: Heatmap results with additional melanoma cells, with individual plots shown in Fig. S4 G. **(C)** The indicated melanoma cells were treated with AZD3514 (5 and 10 µM) versus vehicle control (DMSO) followed by cell density determination by crystal violent staining 7 d later. Data are shown as mean ± SD; one-way ANOVA with Dunnett’s test. Cultures, *n* = 3. ****, P < 0.001. **(D)** In vitro cancer/stromal cell expansion assays, with RFP-expressing A375 melanoma cells cocultured with GPF-expressing HDFs with or without treatment with AZD3514 (10 µM) or DMSO control for 4 d. Shown are representative images and quantification of melanoma cell expansion (percentage area covered by melanoma cells per field). Each dot represents one analyzed field. Number of fields, *n* = 12; two-tailed paired *t* test, ****, P < 0.001. Scale bar: 30 µm. **(E)** Proliferation live-cell imaging assays (IncuCyte) of two primary melanocyte strains cultured in medium with charcoal-stripped serum and treated with DHT at the indicated concentrations versus DMSO control. Number of wells, *n* = 3; Pearson r correlation test. *, P < 0.05; **, P < 0.01. Results of a similar assay with another primary melanocyte strain and melanoma cells are shown in Fig. S4 (I and K). **(F)** Cell density assays (CellTiter-Glo) of WM1366 melanoma cells in medium with charcoal-treated serum and treated with DHT (20 nM) versus DMSO control for the indicated days. Data are shown as mean ± SD; one-way ANOVA with Dunnett’s test. Cultures, *n* = 9, all experiments repeated three times. *, P < 0.05. Right: Heatmap results with additional melanoma cells, with individual plots shown in Fig. S4 J.

AR is a fundamental target for therapy of metastatic prostate cancer, and inhibitors with multiple mechanisms of action and efficacy have been developed (Crawford et al., 2018). Treatment of different melanoma cell lines with several AR inhibitors, including one that functions through both ligand-competitive and noncompetitive mechanisms, AZD3514 (Loddick et al., 2013), and another, pure ligand competitive inhibitor, enzalutamide (Bambury and Scher, 2015), exerted similar growth-suppressive effects, although at different doses (Fig. 3 A). The first compound exhibited a greater potency, which we found to be associated, as previously reported for LNCaP cells (Loddick et al., 2013), with down-modulation of AR expression in two of three tested cell lines (Fig. S4 F). The AZD3514 inhibitory effects were confirmed by treatment of a larger panel of melanoma cell lines and primary melanoma cells with different levels of AR expression, consistent with the basal protective function investigated below (Fig. 3, B and C; and Fig. S4, G and H).

We recently reported that suppression of AR activity in human dermal fibroblasts (HDFs) by a ligand-competitive inhibitor induces expression of a battery of tumor-promoting cancer-associated fibroblast effector genes, similarly to silencing of the gene (Clocchiatti et al., 2018). To assess the net effects of AR inhibitors on melanoma cells in the presence of surrounding HDFs, we used an in vitro cancer/stromal cell expansion assay based on the coculture in Matrigel of fluorescently labeled cells (Clocchiatti et al., 2018). As shown in Fig. 3 D, expansion of melanoma cells admixed with HDFs was significantly reduced by treatment with the AR inhibitor AZD3514, consistent with the efficacy of this compound in the in vivo assays shown further below.

Conversely to the growth suppressing effects of the AR inhibitors, proliferation of primary melanocytes and melanoma cells in charcoal-stripped medium was significantly enhanced by treatment with the AR agonist dihydrotestosterone (DHT) in a dose-dependent manner (Fig. 3 E and Fig. S4 I). Proliferation of other melanoma cell lines and primary melanoma cells in charcoal-stripped medium was also enhanced by DHT stimulation (Fig. 3 F and Fig. S4, J and K) and, when they were cultured under very sparse conditions, their expansion was very highly dependent on the hormone (Fig. S4 L). Thus, besides being required, increased AR signaling is a positive determinant of melanoma cell proliferation.

### The melanoma AR-dependent gene signature is of clinical relevance

**Figure 4.**
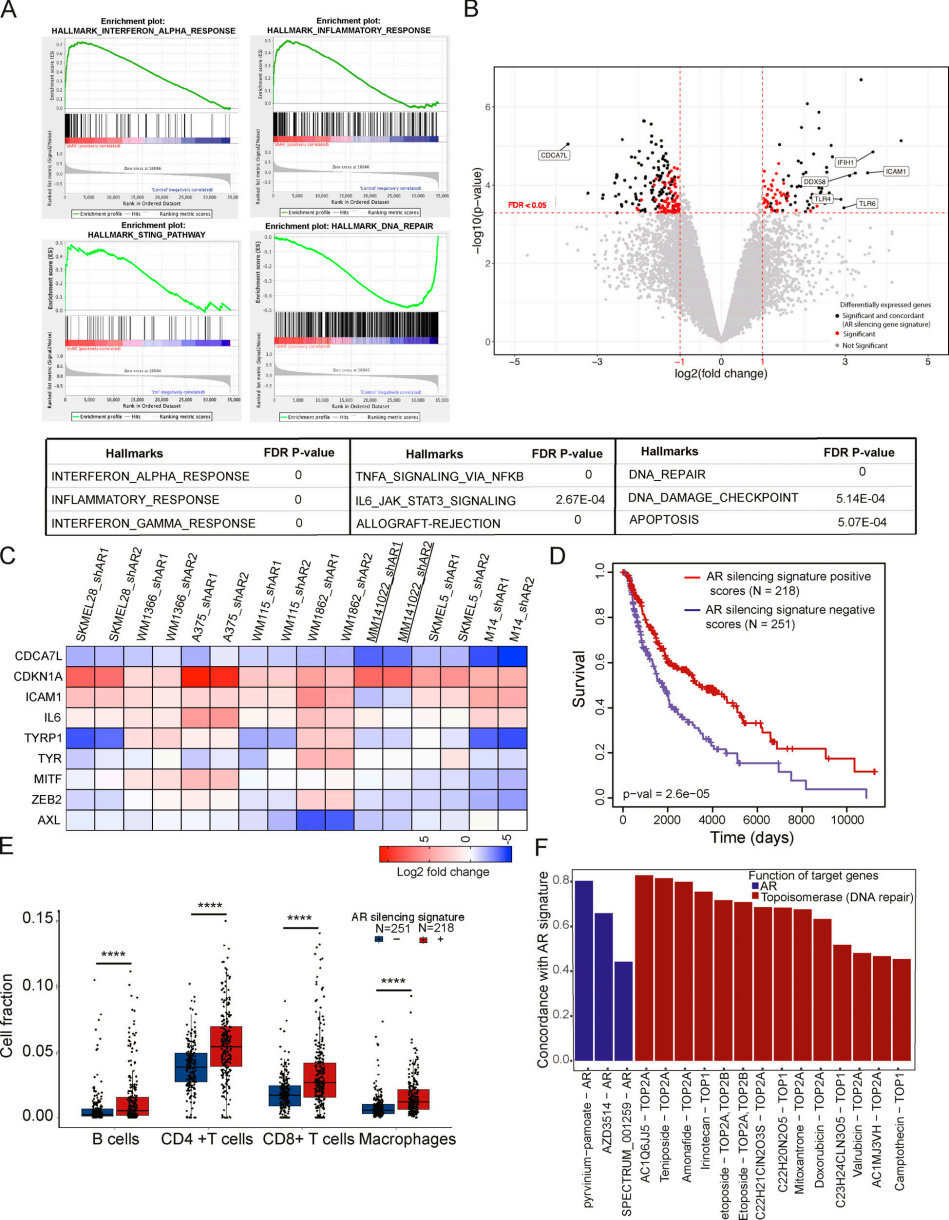
**Global analysis of AR-regulated genes in melanoma cells and clinical relevance. (A)** GSEA of transcriptional profiles elicited by *AR* silencing in WM1366, SKMEL28, and WM115 melanoma cells by two different lentiviruses versus empty vector control, using a predefined set of gene signatures related to cellular processes and signaling pathways (Broad Institute, http://software.broadinstitute.org/gsea/msigdb/collections.jsp#H). Cells were analyzed 5 d after infection by Clariom D array hybridization. Top: Plot distribution of gene signatures related to IFNα, inflammatory response, and DNA repair pathways. Genes are ranked by signal-to-noise ratio in *AR*-silenced versus control melanoma cells; position of individual genes is indicated by black vertical bars; enrichment score is in green. Bottom: Relevant gene sets most significantly associated with *AR* silencing gene signature are indicated together with the corresponding false discovery rate q values. The full list of significantly associated gene signatures is provided in Table S4. **(B)** Volcano plot of shared transcriptional changes in WM1366, SKMEL28, and WM115 melanoma cells with or without *AR* silencing. The x axis shows the log2 fold-change, and the y axis shows −log10 of statistical significance (P value). A false discovery rate threshold of 0.05 and fold-change thresholds of −1 and 1 are indicated by dashed red lines. Each dot represents one gene. Gray and red dots correspond to genes not significantly or nonconcordantly modulated in the three melanoma lines, respectively. Black dots show genes above thresholds that are concordantly up- or down-regulated in all three cell lines and compose the *AR*-silencing gene signature used for further analysis. A few selected genes among the most significantly differentially expressed ones are indicated. The list of 155 genes associated with *AR*-silencing gene signature is provided in Table S3. **(C)** Expression of the indicated genes in multiple melanoma cell lines with or without *AR* silencing by two different lentiviruses versus empty vector control. **(D)** Association of the *AR*-silencing gene signature in melanoma cells (as obtained in B) with patients’ survival in SKCM dataset. Positive and negative association scores for each patient were computed from RNA-sequencing data with GSVA R package. Kaplan–Meier curves show that melanomas with positive association with the *AR*-silencing signature (red, *n* = 251) have better survival than those with negative association (blue, *n* = 218); P = 2.6 × 10^−5^, log-rank test. **(E)** Fraction of tumor-infiltrating immune cells estimated by EPIC R package analysis of SKCM dataset, using default reference profile in tumors with positive and negative association with the *AR*-silencing signature (red and blue box plots, respectively). Cell fractions for B cells, CD4^+^ T cells, CD8^+^ T cells, and macrophages are reported (each dot representing one tumor). Outliers with cell fraction >0.15 are not shown. The additional enrichment scores of signature matrix associated with 22 different immune cell types determined by CIBERSORTx are shown in Fig. S5 A (nonsignificant subpopulations are not shown). ****, P < 0.001. **(F)** Bar plot reporting the concordance between the melanoma *AR*-silencing gene signature and iLINCS expression profiles of A375 cells treated with compounds targeting AR (blue), TOPO1, and TOPO2A (red). Perturbagens of each class are sorted by concordance (P < 0.0001), and names of chemical compounds are reported on the x axis along with molecular targets. A list of compounds eliciting gene expression profiles with concordance coefficient >0.6 with *AR*-silencing signature is reported in Table S5.

AR controls transcription through both direct and indirect DNA binding mechanisms (Matsumoto et al., 2013). We performed transcriptomic analysis of three different melanoma lines, two with *BRAF* and one with *NRAS* mutations, with or without *AR* silencing with two different lentiviruses. By gene set enrichment analysis (GSEA; Subramanian et al., 2005), gene signatures related to IFN-, cytokine-, and STING-signaling pathways were the most significantly associated with the gene expression profiles resulting from *AR* silencing (Fig. 4 A and Table S4) together with those related to DNA repair and apoptosis.

Next, we established an *AR* gene-silencing signature of 155 genes, which were significantly and concordantly modulated by *AR* silencing in all three melanoma cell lines (Fig. 4 B and Table S3). The most down-modulated gene was *CDCA7L*, coding for a transcriptional repressor and c-MYC interacting protein with shared oncogenic function (Hendrix et al., 2014; Tian et al., 2013), while the most up-regulated genes included several with key immunomodulatory functions, such as *ICAM1* (Adutler-Lieber et al., 2018), *TLR4* and *TLR6* (Rakoff-Nahoum and Medzhitov, 2009), *DDX58* (RIG-1), and *IFIH1* (melanoma differentiation–associated factor 5; Brisse and Ly, 2019; Fig. 4 B and Table S3).

The analysis was extended to a panel of other melanoma cell lines and primary melanoma cells with different levels of *AR* expression by RT-qPCR. *CDCA7L* expression was down-modulated while *CDKN1A* was up-regulated in all cells by *AR* silencing, consistent with the observed induction of cellular senescence. Intercellular adhesion molecule 1 (ICAM1) was consistently up-regulated together with IL6, a potent proinflammatory cytokine (Fig. 4 C). As for “canonical” genes involved in melanoma progression, differentiation marker genes such as *TyR* and *TYRP1* were either up- or down-modulated by *AR* silencing in the various cell lines, and so were the *MITF* master regulatory gene (Roider and Fisher, 2014) and *ZEB2*, coding for a transcription factor with a role in melanogenesis upstream of MITF expression (Denecker et al., 2014). *AXL*, coding for a receptor tyrosine kinase implicated in melanoma aggressive behavior (Revach et al., 2019), was mostly down-modulated (Fig. 4 C).

**Figure S5.**
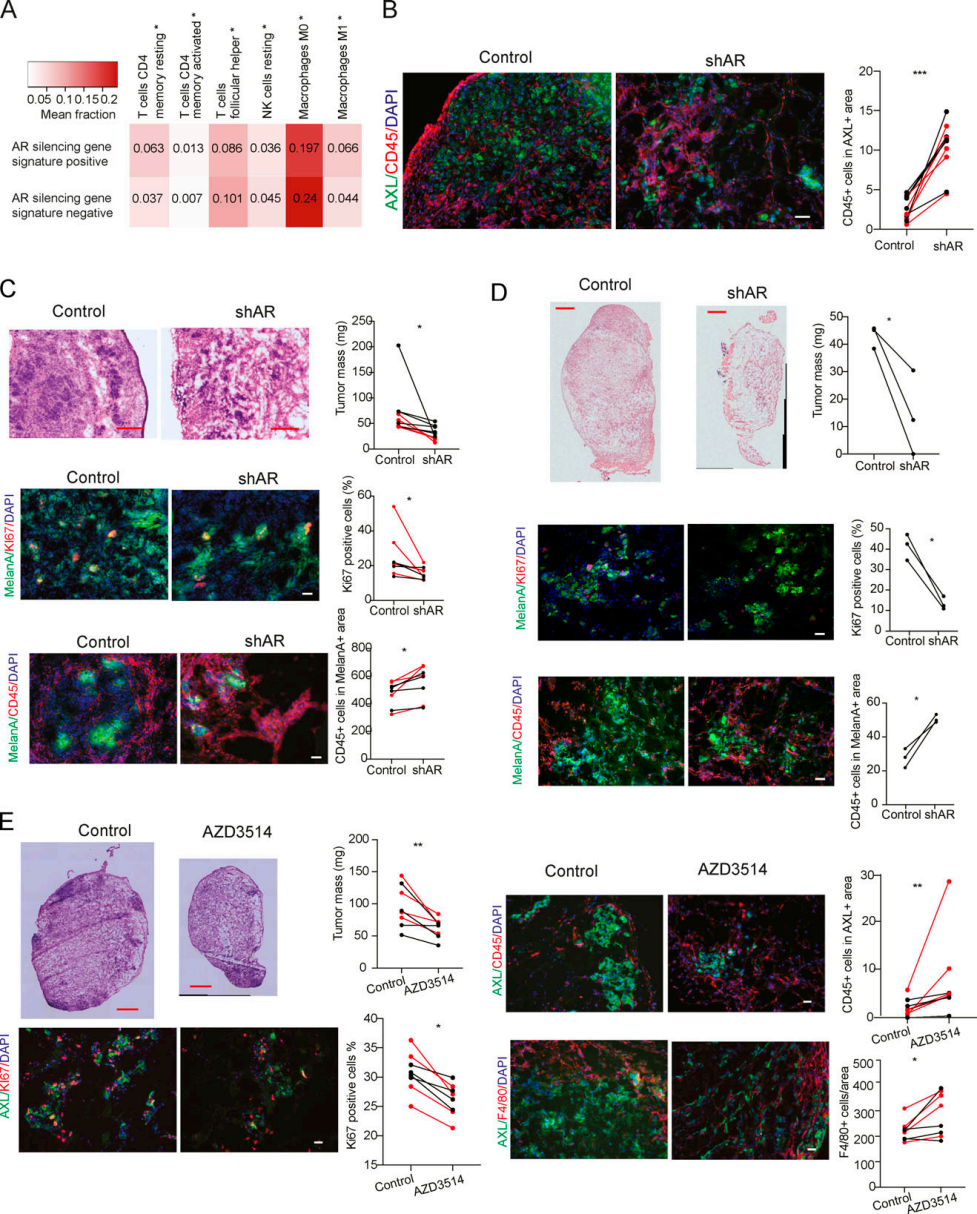
**Suppression of melanoma formation by *AR* silencing or inhibition. (A)** Prevalence of stromal and immune cells in TCGA SKCM samples with and without enrichment for the *AR*-silencing gene signature. Heatmaps reporting mean fractions of significantly prevalent (Wilcoxon rank-sum test, Bonferroni-adjusted P < 0.05) stromal and immune cell types (columns) for TCGA SKCM samples with *AR*-silencing signature up or down (rows) obtained using CIBERSORTx. Red intensity is proportional to the mean cell fraction, which is also reported in each entry. **(B)** Double IF analysis of lesions from Fig. 8 A with antibodies against AXL, for melanoma cell identification and CD45-positive cells. Shown is the quantification together with representative images of CD45-positive cells per AXL-positive tumor area, counting in each five fields, five male mice and five female mice; data of male mice in red. Scale bars: 20 µm. ***, P < 0.005. **(C)** *AR* silencing inhibits A375 melanoma tumorigenesis. Top: Tumor size, measured by digital caliper (mass = [length × width × height] × π/6) together with representative low-magnification H&E images of the retrieved lesions. Middle: Double IF analysis of lesions with antibodies against MelanA (green), for melanoma cell identification, and Ki67-positive cells. Shown are representative images of MelanA-positive cells stained with antibodies against the other markers, together with relative quantification (counting in each case >50 cells in three to five fields on digitally retrieved images, using ImageJ). Bottom: Double IF analysis of lesions with antibodies against MelanA, CD45, for melanoma cells, and hematopoietic cell identification, respectively. Shown are representative images together with quantification of number of F4/80-positive cells per MelanA-positive tumor area, counting in each case three to four fields. Control versus experimental lesions, *n* = 20; two-tailed paired *t* test, *, P < 0.05; **, P < 0.01; ***, P < 0.005. Scale bars: 10 µm. **(D)** *AR* silencing inhibits SKMEL28 melanoma tumorigenesis. Top: Tumor size, measured by digital caliper (mass = [length × width × height] × π/6) together with representative low-magnification H&E images of the retrieved lesions. Scale bars: 100 µm. Middle: Double IF analysis of lesions with antibodies against MelanA (green), for melanoma cell identification, and Ki67-positive cells. Shown are representative images of MelanA-positive cells stained with antibodies against the other markers, together with relative quantification (counting in each case >50 cells in three to five fields on digitally retrieved images, using ImageJ). Bottom: Double IF analysis of lesions with antibodies against MelanA, CD45 for melanoma cells, and hematopoietic cell identification, respectively. Shown are representative images together with quantification of number of CD45-positive cells per MelanA-positive tumor area, counting in each case three to four fields. Control versus experimental lesions, *n* = 6; two-tailed paired *t* test, *, P < 0.05. Scale bars: 10 µm. **(E)** AZD3514 pretreatment inhibits WM1366 melanoma tumorigenesis. Top left: Tumor size, measured by digital caliper (mass = [length × width × height] × π/6) together with representative low-magnification H&E images of the retrieved lesions. Double IF analysis of lesions with antibodies against AXL (green), for melanoma cell identification, and Ki67-positive cells (lower left). Shown are representative images of AXL-positive cells stained with Ki67 together with relative quantification (counting in each case >50 cells in three to five fields on digitallyretrieved images, using ImageJ). Right: Double IF analysis of lesions with antibodies against AXL, CD45, and F4/80, for melanoma cell, hematopoietic cell, and macrophage identification, respectively. Shown are representative images together with quantification of number of F4/80-positive cells per AXL positive tumor area (counting in each case three to four fields). Control versus experimental lesions, *n* = 16; two-tailed paired *t* test, *, P < 0.05; **, P < 0.01; ***, P < 0.005. Scale bars: 10 µm.

To assess the clinical significance of the findings, we examined the gene expression profiles of a cohort of 469 cutaneous melanomas in the Cancer Genome Atlas (TCGA) dataset Skin Cutaneous Melanoma (SKCM). Tumors were stratified as having positive or negative association scores with the *AR* gene-silencing signature that we established. Tumors with positive scores had significantly higher patient survival than those with negative scores (log-rank test, P = 2.6 × 10^−5^; Fig. 4 D). The findings remained significant after correcting for age, sex, genomic subtype, and primary or metastatic status (multivariate Cox regression, P = 0.002). Analysis of the transcriptomic profiles by the Epic algorithm (Racle et al., 2017) was used to estimate the proportion of cancer-infiltrating immune cells. A significantly higher proportion of infiltrating B cells, CD4^+^ and CD8^+^ T cells, and macrophages was found in melanomas with a positive association with the *AR* gene-silencing signature than in those with a negative association (Fig. 4 E). The results were validated and refined with an independent approach, CIBERSORTx (Newman et al., 2019), showing that tumors with a positive association with the *AR* silencing signature were selectively enriched for M1-like versus M2-like macrophages, and for CD4^+^ memory T cells (Fig. S5 A).

### AR loss triggers genomic DNA breakage, cytoplasmic leakage, and STING-dependent gene expression

**Figure 5.**
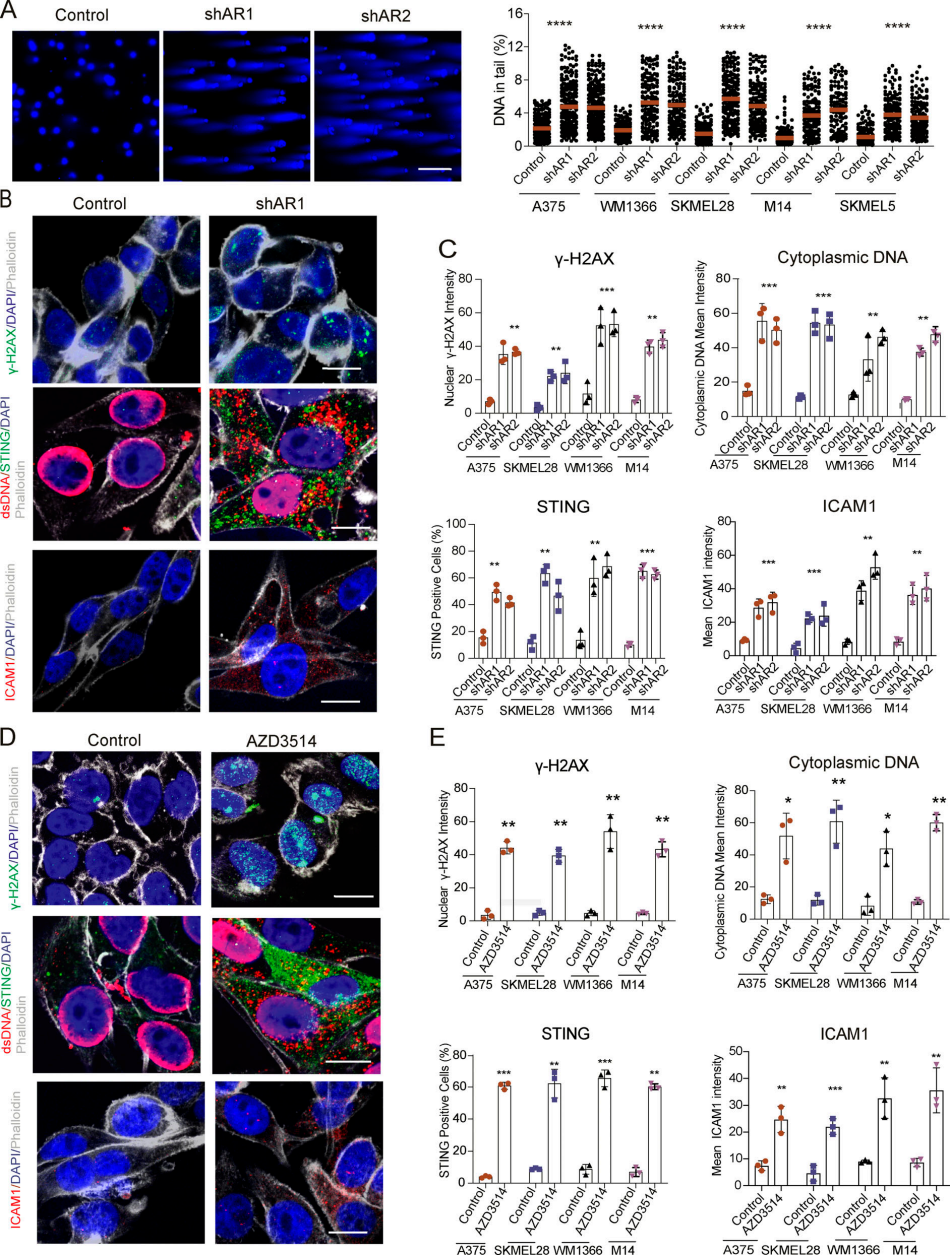
**Loss of AR function induces DNA breakage, cytoplasmic dsDNA leakage, and STING activation. (A)** Comet assays of melanoma cell lines with or without *AR* silencing on day 1 after selection. Shown are representative images of WM1366 melanoma cells together with quantification of percentage tail DNA (Comet Score) in five different melanoma cell lines. Scale bar: 10 µm. Number of cells, *n* =125; one-way ANOVA; ****, P < 0.001. **(B)** Representative double IF images of WM1366 cells with or without *AR* silencing stained with antibodies against γ-H2AX (green) and phalloidin (gray; upper panel), dsDNA (red) and STING (green; middle panel), and ICAM1 (red; lower panels). Scale bar: 10 µm. **(C)** Quantification of nuclear γ-H2AX, cytoplasmic DNA, ICAM1 IF signal intensity, and percentage of STING-positive cells in the indicated panel of melanoma cell lines with or without *AR* silencing. More than 100 cells were counted in each condition. Results are expressed as mean ± SD. Cultures, *n* = 3; one-way ANOVA with Dunnett’s test, **, P < 0.01, ***, P < 0.005. **(D and E)** Double IF image analysis of a panel of melanoma cells treated with AZD3514 (10 µM) versus DMSO control for 2 d. Shown are representative images (D) and quantification (E) of the results as in C. Cultures, *n* = 3; two-tailed paired *t* test, *, P < 0.05; **, P < 0.01, ***, P < 0.005.

**Figure 6.**
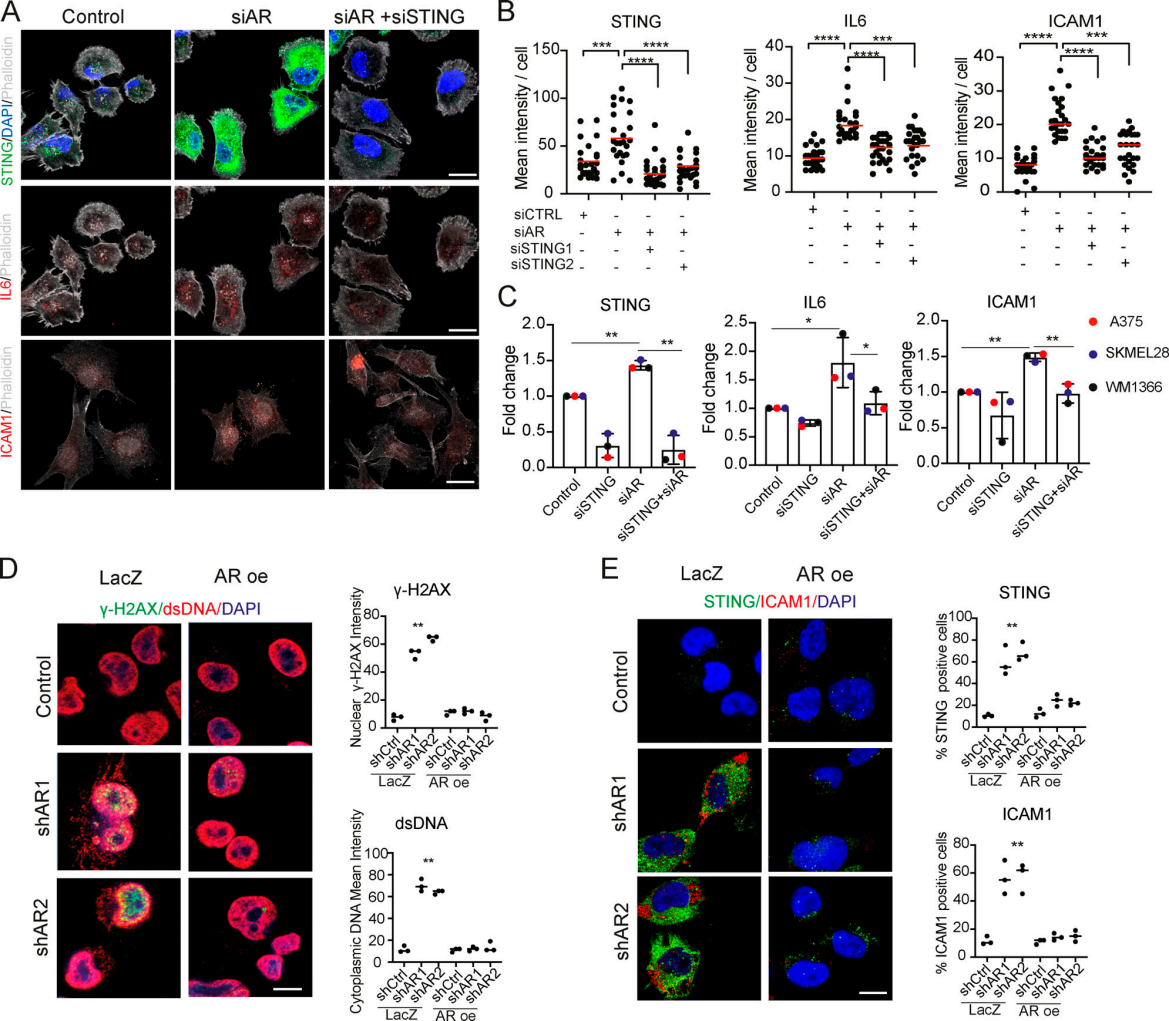
**Loss of AR function induces STING-dependent gene expression. (A and B)** Double IF analysis of WM1366 melanoma cells transfected with *STING* and/or *AR*-silencing siRNAs versus scrambled controls, with antibodies against STING (upper panel, green), IL6, and ICAM1 (middle and lower panels, red) and phalloidin staining for cell border delimitation (gray). Shown are representative images (A) and quantification (B) of STING, IL6, and ICAM1 fluorescence signal intensity per cell, 48 h after transfection. Each dot corresponds to mean fluorescence intensity per cell. Number of cells, *n* = 25; paired *t* test, ***, P < 0.005, ****, P < 0.001. Scale bar: 10 µm. **(C)** RT-qPCR analysis of STING, IL6, and ICAM1 mRNA expression in the indicated melanoma cell lines 48 h after transfection with *STING* and/or *AR*-silencing siRNAs versus scrambled controls. Each bar corresponds to mean expression levels per melanoma cell line. Data are represented as mean ± SD. Number of strains, *n* = 3; one-way ANOVA with Dunnett’s test, *, P < 0.05, **, P < 0.01. **(D) **Representative double IF images and quantification of γ-H2AX expression (green) and cytoplasmic dsDNA leakage (red) in A375 cells stably infected with an AR-overexpressing (AR oe) or control lentivirus and superinfected with two *AR*-silencing lentiviruses versus control. Scale bar: 10 µm. Data are from triplicate experiments; each dot represents one experiment. Cultures, *n* = 3; one-way ANOVA with Dunnett’s test, **, P < 0.01. **(E)** Representative double IF images and quantification of STING (green) and ICAM1 (red) expression in A375 cells with or without AR overexpression and silencing as in D. Independent experiments, *n* = 3; one-way ANOVA with Dunnett’s test, **, P < 0.01.

The iLINCS (Integrative LINCS; http://www.ilincs.org/ilincs/) portal allows comparative analysis of transcriptional profiles of various cell lines in response to different drugs. A significant concordance was found between the *AR* silencing gene signature and the iLINCS-derived transcriptional profiles of A375 melanoma cells treated with several AR inhibitors, as well as a number of DNA-damaging agents targeting the topoisomerase 2 (TOPO2) and TOPO1 enzymes (Fig. 4 F and Table S5). Consistent with these findings, comet assays showed that *AR* gene silencing was sufficient to induce massive chromosomal DNA breakage in several melanoma cells, irrespective of endogenous levels of AR expression (Fig. 5 A), which was accompanied by induction of γ-H2AX, a marker of the DNA damage response (Bonner et al., 2008; Fig. 5, B and C). In parallel, *AR* silencing resulted in the abundant release of double-stranded DNA (dsDNA) fragments into the cytoplasm, together with increased expression and aggregation of the STING protein, a cytosolic DNA sensor with an important role in innate immunity (Chen et al., 2016; Fig. 5, B and C). Similar observations were also obtained by CRISPRi downmodulation of AR expression and treatment with the AR inhibitor AZD3514 (Fig. 5, D and E; and Fig. S4, M and N). The findings are of functional significance, as induction of IL6 and ICAM1, two STING target genes with key immune-modulatory functions (Chen et al., 2016), was suppressed at both protein and mRNA levels by concomitant *AR* and *STING* knockdown (Fig. 6, A–C). The link between AR loss and ensuing events was further supported in that chromosomal DNA damage and leakage into the cytoplasm, STING activation, and IL6 and ICAM1 induction were all suppressed in cells in which *AR* gene silencing was counteracted by overexpression (Fig. 6, D and E).

### AR plays an essential function in anchoring the Ku70 and Ku80 DNA repair proteins to RNA Pol II and preventing RNA Pol II–associated DNA damage

**Figure 7.**
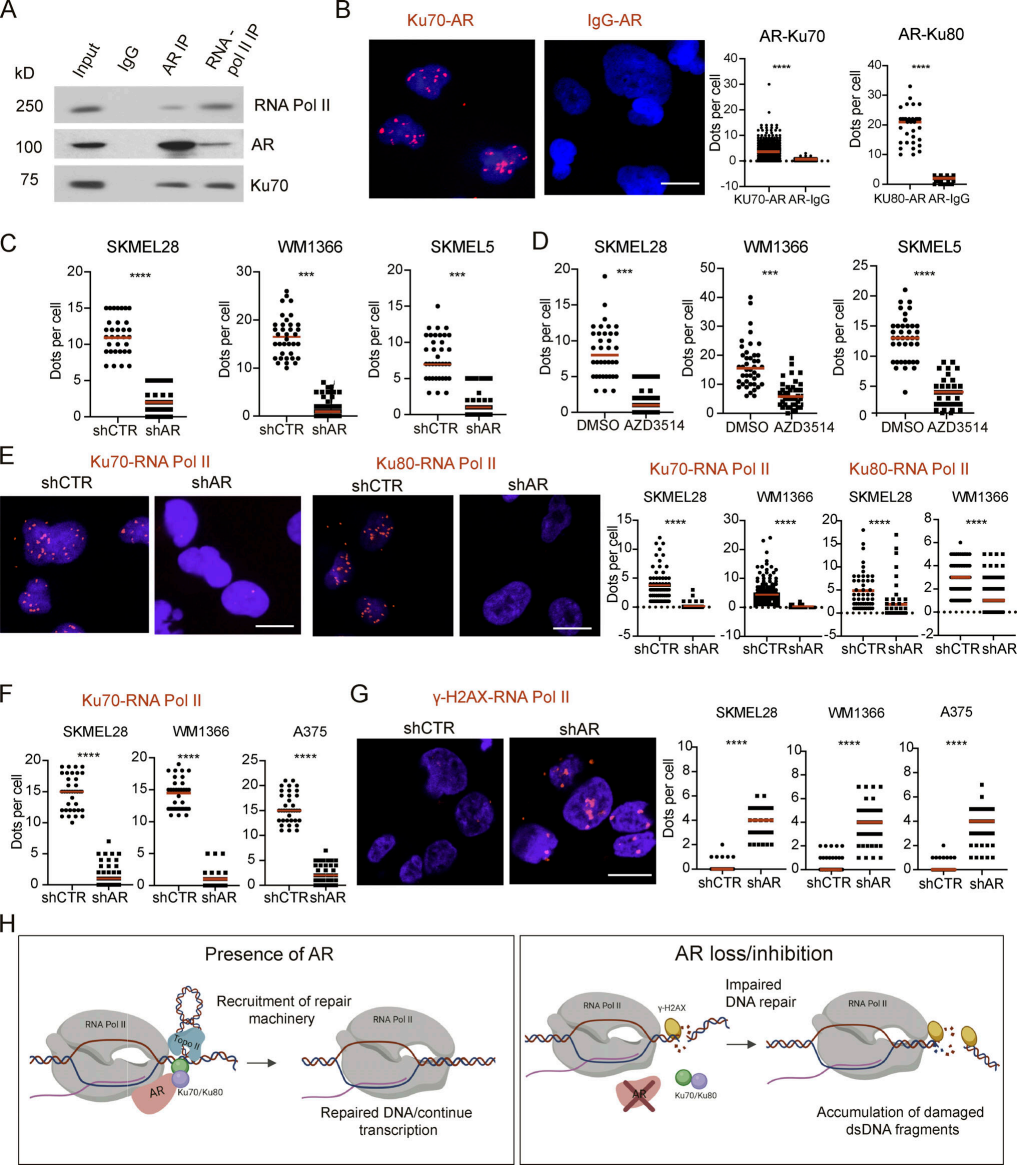
**AR anchors the Ku70 and Ku80 DNA repair proteins to RNA Pol II and prevents RNA Pol II–associated DNA damage. (A)** Co-IP analysis with anti-AR and anti-RNA pol II antibodies in WM1366 melanoma cells and immunoblotted for indicted proteins. **(B)** PLAs of WM1366 melanoma cells with antibodies against AR and Ku70 and nonimmune IgGs as specificity control. Red fluorescence puncta resulting from the juxtaposition of anti-AR and anti-Ku70/Ku80 antibodies were visualized by confocal microscope with concomitant DAPI nuclear staining. Shown are representative images and quantification of the number of puncta per cell. For this and following panels, *n* (cells per condition) > 50; ***, P < 0.005; ****, P < 0.001, 2-tailed unpaired *t* test. **(C)** PLAs of AR and Ku70 association in melanoma cell lines with elevated (WM1366 and SKMEL28) versus low (SKMEL5) levels of total AR protein (as shown in Fig. 1 E), with or without shRNA-mediated *AR* gene silencing. ***, P < 0.005; ****, P < 0.001. **(D)** PLAs of AR and Ku70 association in the same melanoma cell lines as in C with or without treatment with the AR inhibitor AZD3514 (10 µM for 48 h). ***, P < 0.005; ****, P < 0.001. **(E and F)** PLAs of the indicated melanoma cell lines with or without *AR* gene silencing with antibodies against Ku70 or Ku80 and total RNA Pol II (E) or elongating form (CTD Ser2 phosphorylated; F). ****, P < 0.001. **(G)** PLAs of melanoma cells with or without *AR* gene silencing as in F, with antibodies against the elongating form of RNA Pol II and γ-H2AX. Shown are better representative images. Scale bars: 10 µm. ****, P < 0.001. **(H)** Diagrammatic model of the AR anchoring function, required for Ku70/Ku80 association to the RNA Pol II transcription complex and prevention of transcription-associated DNA damage.

A number of indirect mechanisms could be responsible for chromosomal DNA breakage resulting from loss of AR activity. However, an attractive possibility is that AR also physically associates with proteins involved in the maintenance of genomic integrity and contributes to their function. A significant amount of endogenous DNA damage occurs in cells in association with gene transcription (Tubbs and Nussenzweig, 2017). The processive function of RNA Pol II is coupled with the release of dsDNA torsional stress by TOPO-mediated cleavage and resealing, with the association of DNA repair proteins such as Ku70 and Ku80 as part of a protective mechanism (Tubbs and Nussenzweig, 2017). Direct binding to Ku70 and Ku80 to AR has been previously reported (Mayeur et al., 2005), raising the attractive possibility that AR is involved in anchoring Ku70 and Ku80 to the transcription apparatus. Coimmunoprecipitation (co-IP) and proximity ligation assays (PLAs) with antibodies against AR and these proteins showed that they effectively associate in various melanoma cell lines (Fig. 7, A and B). A similar number of PLA complexes were detected in cells with different total AR protein levels, with PLA signal being abolished by shRNA-mediated *AR* gene silencing as well as treatment with the AR inhibitor AZD3514, supporting the specificity of the assays (Fig. 7, C and D). Complexes of Ku70 and Ku80 with RNA Pol II were detected by PLA assays with antibodies against total RNA Pol II, as well as specific for the Pol II phosphorylated form (CTD, Ser2) involved in transcription elongation (Phatnani and Greenleaf, 2006; Fig. 7 E, F). Importantly, the association of Ku70 and Ku80 with Pol II was drastically reduced by *AR* gene silencing, pointing to an essential anchoring function of the AR protein (Fig. 7, E and F). Loss of Ku70–RNA Pol II association in cells with loss of AR was mirrored by a drastic increase in foci of DNA damage associated with RNA Pol II, as detected by PLA assays with antibodies against γ-H2AX (Fig. 7 G). Overall, these findings are consistent with a model whereby loss of AR results in detachment of Ku70/Ku80 DNA repair proteins from the RNA Pol II complex and increased dsDNA damage at sites of transcription (Fig. 7 H). This, in combination with additional, more indirect mechanisms, leads to massive dsDNA breakage and leakage into the cytoplasm with activation of the STING-dependent signaling cascade.

### AR loss or inhibition results in reduced tumorigenicity with enhanced immune cell infiltration

**Figure 8.**
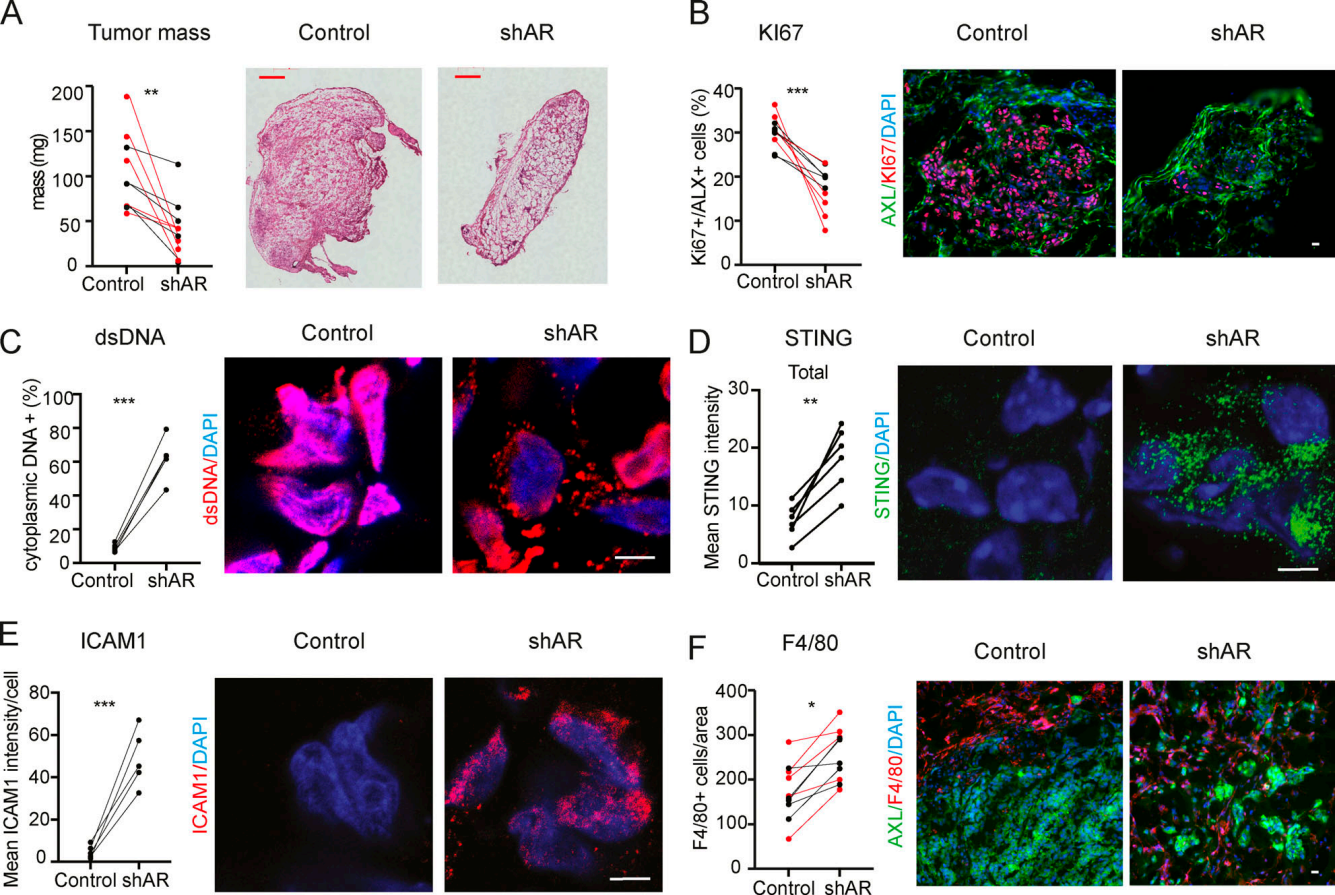
**Suppression of melanoma formation by *AR* silencing.** WM1366 melanoma cells with or without *AR* silencing were tested by parallel intradermal Matrigel injections into NOD/SCID male and female mice (five per group; data of male mice in red). **(A)** Tumor size, measured by digital caliper (mass = [length × width × height] × π/6) together with representative H&E images of the retrieved lesions 16 d after injection. **(B–E)** Double IF analysis of lesions with antibodies against AXL, for melanoma cell identification, quantification of KI67-positive (B) or cytoplasmic dsDNA–positive (C) cells, and mean fluorescence signal intensity of STING (D) and ICAM1 expression (E). Shown are representative images of AXL-positive cells (AXL signal not shown in C–E) stained with antibodies against the other markers, together with relative quantification (>50 cells in three to five fields). **(F)** Representative images and quantification of the number of F4/80-positive macrophages per AXL-positive tumor area, counting in each case three to four fields. Similar determination of CD45 positive cells is shown in Fig. S5 B. Control versus experimental lesions, *n* = 10; two-tailed paired *t* test, *, P < 0.05; **, P < 0.01; ***, P < 0.005. Scale bar: 10 µm. Similar tumorigenicity experiments with A375 and SKMEL28 cells with or without shRNA-mediated *AR* silencing is shown in Fig. S5 (C and D).

To assess the in vivo significance of the findings, we used an orthotopic model of melanoma formation based on the intradermal injection of melanoma cells embedded in Matrigel (Clocchiatti et al., 2018), which enables the assessment of early steps of tumor formation and expansion. Using this assay, we found that tumorigenic expansion and proliferative activity of multiple melanoma cell lines (WM1366, A375, and SKMEL28) was significantly reduced, in male and female mice, by *AR* silencing (Fig. 8, A and B; and Fig. S5, B and C). In parallel, *AR* silencing resulted in cytoplasmic dsDNA release, STING aggregation, and ICAM1 induction (Fig. 8, C–E). While host macrophages were mostly absent in tumors formed by control cells, they actively infiltrated tumors formed by cells with silenced *AR* (Fig. 8 F and Fig. S5, B and C).

**Figure 9.**
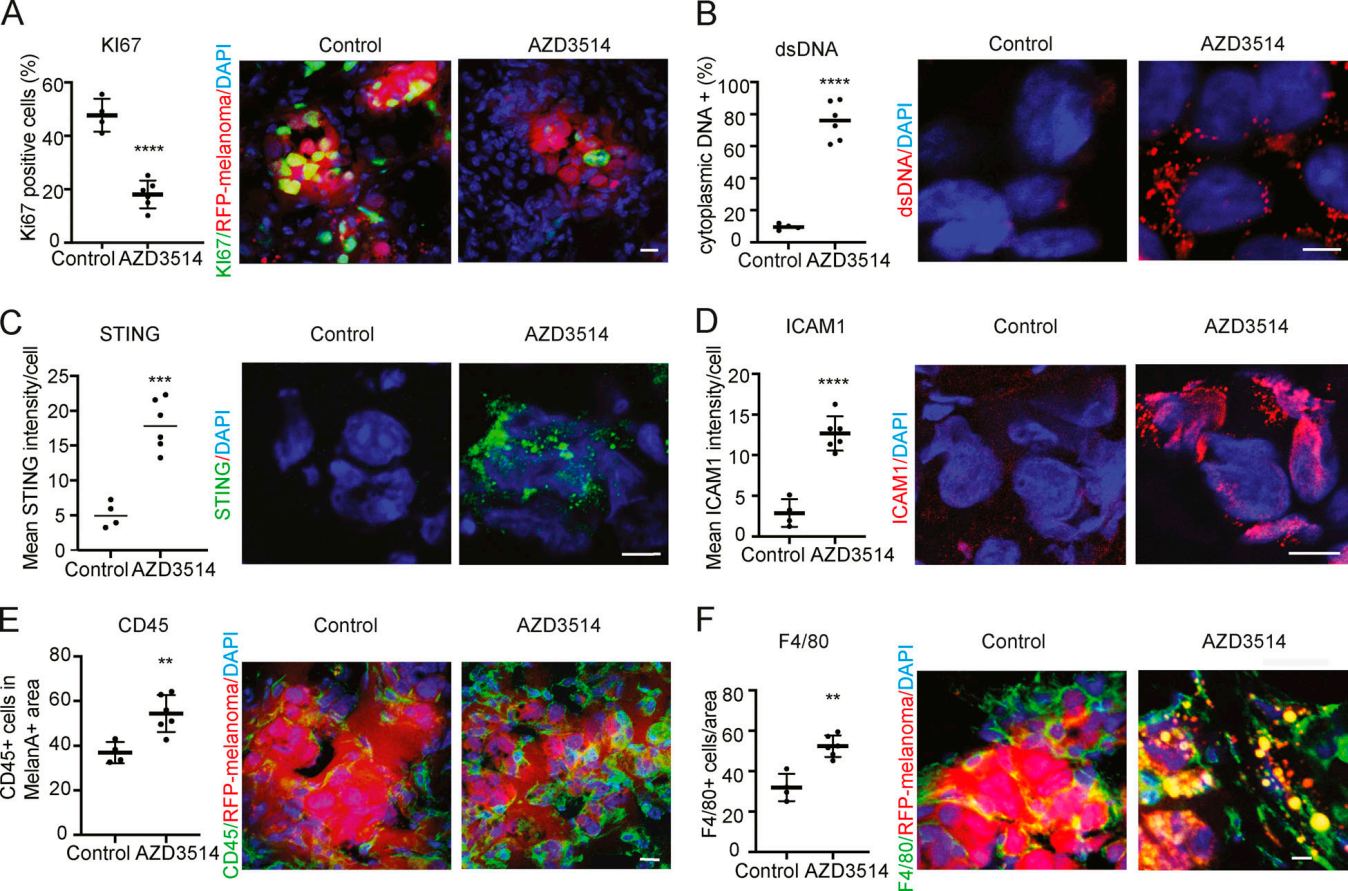
**Suppression of melanoma formation by AR inhibition. (A–F)** RFP-expressing A375 melanoma cells were injected intradermally into 10 male mice. 3 d after injection, mice were treated by oral gavage with either AZD3514 (50 mg/kg) or DMSO vehicle alone for 12 consecutive days. IF analysis was used to assess KI67 (A) and cytoplasmic dsDNA (B) positivity and STING (C) and ICAM1 (D) expression levels in melanoma cells (RFP-positive) together with numbers of juxtaposed leukocytes (E) and macrophages (F), as assessed by staining for the CD45 and F4/80 markers, respectively. Shown are quantifications together with representative images, including one (F) showing engulfment of fragmented RFP-positive melanoma cells into F4/80-positive macrophages in lesions of mice treated with the AZD3514 inhibitor. Control versus experimental lesions, *n* = 4 and 10; unpaired *t* test, **, P < 0.01; ***, P < 0.005, ****, P < 0.001. Scale bar: 10 µm (A–F). Similar tumorigenicity experiments with injection of AZD3514-pretreated WM1366 cells are shown in Fig. S5 E.

These findings are likely of translational significance, as suppression of tumor cell proliferation, together with dsDNA cytoplasmic release, STING activation, and ICAM1 induction, was also observed by treatment of tumor-bearing animals with the AR inhibitor AZD3514 (Fig. 9, A–D) or pretreatment of cells before injection into the animals (Fig. S5 E). Even in this case, the results were accompanied by increased macrophage infiltration (Fig. 9, E and F).

**Figure 10.**
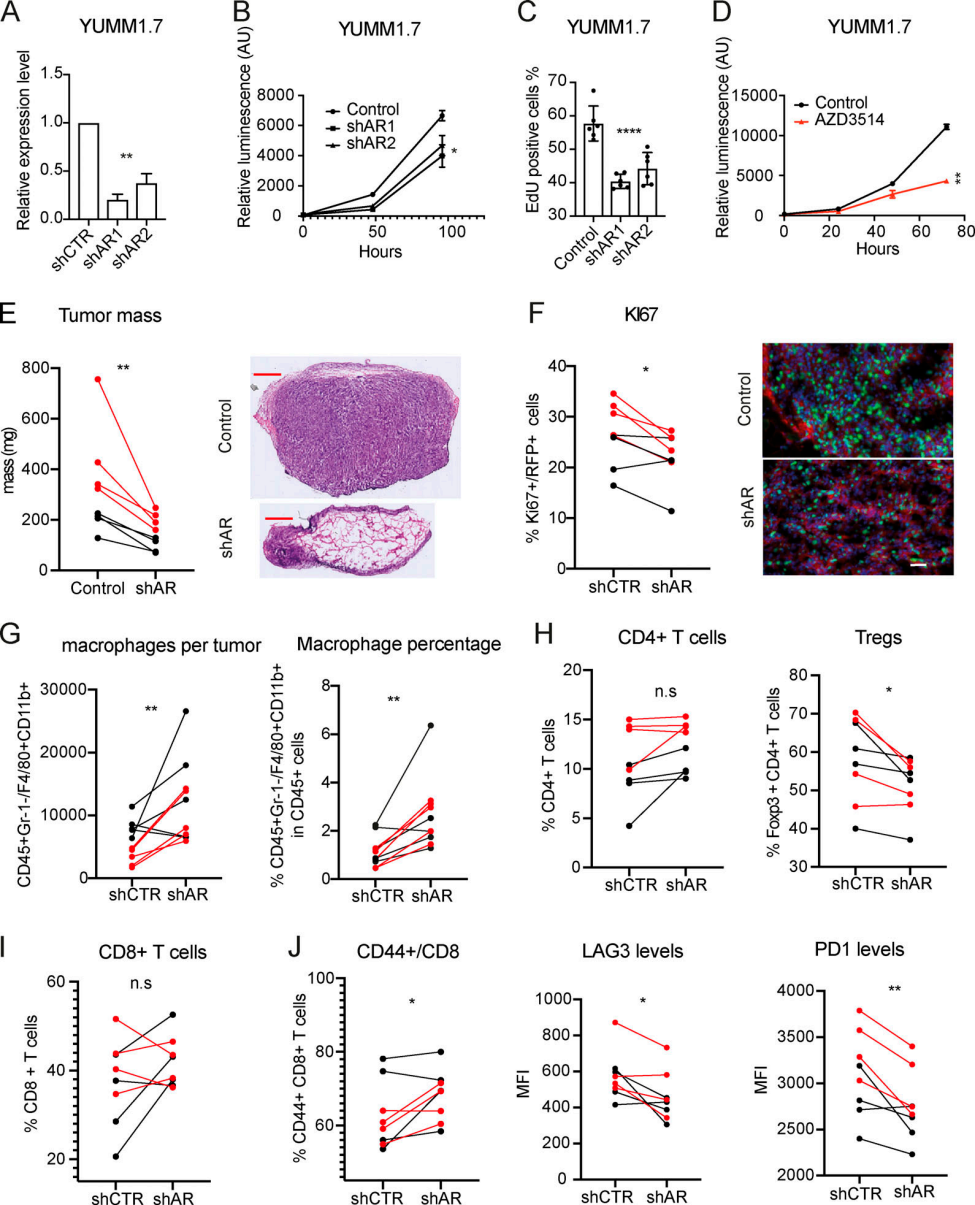
**Suppression of mouse melanoma formation and immune cells recruitment by *AR* gene silencing. (A–C)** Mouse melanoma cells YUMM1.7 were infected with two *AR*-silencing lentiviruses versus empty vector control, 24 h after selection, by determination of *AR* mRNA levels (A), cell density (B), and EdU labeling assay (C). **(D)** YUMM1.7 cells were treated with AZD3514 (10 µM) versus DMSO control followed by cell density assays on the indicated days after treatment. Data are shown as mean ± SD; one-way ANOVA with Dunnett’s test, *, P < 0.05; **, P < 0.01; ****, P < 0.001. Cultures, *n* = 6. **(E and F)** RFP-expressing YUMM1.7 cells with or without *AR* silencing were tested by parallel intradermal Matrigel injections into C57BL/6JOIaHsd male and female mice (four per group; data of male mice in red). Shown are representative H&E and IF images of the retrieved lesions 14 d after injections and determination of tumor mass (E) and percentage of KI67-positive RPF-expressing YUMM1.7 cells. For F, >50 cells in three to five fields per lesion; control versus experimental lesions, *n* = 8; two-tailed paired *t* test, *, P < 0.05. Scale bar: 500 µm (E) and 100 µm (F). **(G–J)** FACS analysis of tumor dissociated cells for total numbers of macrophage cells (CD45^+^ Gr-1^−^ F4/80^+^ CD11b^+^; G), percentage of macrophages in the CD45^+^ cell populations (left and right panels, respectively; H), percentage of CD4^+^ T cells (CD45^+^ CD3^+^ CD4^+^) over total CD45^+^ leukocytes and fraction of regulatory T cells (Tregs; CD45^+^ CD3^+^ CD4^+^ FoxP3^+)^ within CD4^+^ T cells, and percentage of CD8^+^ T cells (CD45^+^ CD3^+^ CD8^+^) over total CD45^+^ leukocytes (I) and of CD44^+^ population of CD8^+^ T cells together with mean fluorescence intensity (MFI) levels of LAG-3 and PD-1 staining in CD44^+^ fraction cells (J). Control versus experimental lesions, *n* = 8; two-tailed paired *t* test, *, P < 0.05; **, P < 0.01; n.s, not significant.

To further elucidate whether AR expression can influence the immunogenicity of melanoma cells and the immune infiltrates in the tumor microenvironment (Wu et al., 2020), we used an immunocompetent model system based on the injection of the mouse melanoma cell line YUMM1.7 (Meeth et al., 2016) into syngeneic mice (BL6 strain). Silencing of the mouse *AR* gene by two different shRNA lentiviral vectors or treatment with AR inhibitors resulted in a significant reduction of proliferation, similar to what we observed with the human cells (Fig. 10, A–D).

In vivo, upon intradermal injection into immune-competent mice, melanoma cells with silenced *AR* formed much smaller tumors than controls, with substantially reduced melanoma cell density and proliferative index (Fig. 10, E and F). Dissociation of tumor cells followed by FACS analysis showed a significant increase of macrophages (CD45^+^ Gr-1^−^ F4/80^+^ CD11b^+^) in *AR*-silenced YUMM1.7 melanomas, consistent with what we observed with human cells in immune-compromised mice (Fig. 10 G). While the total number of CD4^+^ T cells (CD45^+^ CD3^+^ CD4^+^) was not significantly different, that of CD4^+^ regulatory T cells (CD45^+^ CD3^+^ CD4^+^ FoxP3^+^) was significantly decreased in in *AR*-silenced YUMM1.7 melanomas (Fig. 10 H). Percentage levels of total CD8^+^ T cells (CD45^+^ CD3^+^ CD8^+^) did not vary consistently; however, the activated fraction (CD44^+^ population) was significantly increased, with a lesser expression of coinhibitory molecules, LAG3 and PD1, which are highly expressed by exhausted T cells (Yang et al., 2017; Fig. 10, I and J). Thus, in a syngeneic mouse model, decreased tumorigenicity of melanoma cells with AR loss is associated with enhanced modulation of innate and acquired immunity.

## Discussion

The impact of sex hormone signaling in cancer development in organs with nonreproductive functions is still poorly understood (Clocchiatti et al., 2016). We have shown here that sustained AR signaling is key for melanoma cell proliferation potential and tumorigenesis in cells from both male and female individuals. Irrespective of its expression levels, AR plays an essential function in preventing cellular senescence and genomic DNA damage. This is in contrast with previous reports on prostate cancer cells, in which AR inhibition, while synthetically lethal with other treatments (Karanika et al., 2017; Li et al., 2017), does not appear to be sufficient to induce DNA damage and downstream events by itself. In fact, a number of reports indicate that DNA damage can be induced in prostate cancer cells by overstimulation of AR activity (Chatterjee et al., 2019; Lin et al., 2009).

A number of convergent mechanisms are likely to be implicated in the AR dependence of melanoma cells, as revealed by shRNA-mediated silencing of the gene, down-modulation by a CRISPRi approach, and pharmacological inhibition by AR inhibitors. Modulation of multiple genes under AR control is likely to be involved in the suppression of proliferation that results from AR loss, including up-regulation of *CDKN1a*, an inducer of cellular senescence and direct AR negative target (Clocchiatti et al., 2018), and down-modulation of *CDCA7L*, coding for a c-MYC–interacting protein with pro-oncogenic function in other cell types (Hendrix et al., 2014; Tian et al., 2013). Opposite modulation of these genes could contribute to the growth-promoting effects exerted by increased AR activity in melanoma cells as well as primary melanocytes. We previously showed that in dermal fibroblasts, as in melanoma cells, loss of AR activity by either gene silencing or pharmacological approaches results in cellular senescence together with a senescence-associated secretory phenotype that promotes tumorigenesis of neighboring cancer cells (Clocchiatti et al., 2018). Thus, AR loss can have a two-edged sword effects on cancer cells versus surrounding stromal cells, while inducing cellular senescence in both. In melanoma development, the consequences on cancer cells are likely prevalent, since in coculture assays of melanoma cells and dermal fibroblasts, a potent AR inhibitor exerted net beneficial effects that paralleled those observed in vivo, in an orthotopic model of melanoma formation.

Besides suppression of proliferation, another major consequence of *AR* gene silencing or pharmacological inhibition was dsDNA breakage in the absence of additional exogenous insults, with dsDNA leakage into the cytoplasm and ensuing STING activation. Together with a number of possible mechanisms related to AR control of gene transcription, we have found that AR plays a more direct and essential role in anchoring the DNA repair proteins Ku70/Ku80 to RNA Pol II and preventing RNA Pol II–associated DNA damage. A similar number of AR-Ku70 and AR-dependent Ku70/80-RNA Pol II complexes could be detected in melanoma cell lines with substantially different total AR levels, suggestive of a minor pool of this protein being selectively involved in Ku70/80 anchoring function. A direct association of Ku70/Ku80 with AR has already been reported for prostate cancer cells, in which, however, Ku70 and Ku80 were implicated in control of AR-dependent transcription (Mayeur et al., 2005), rather than AR being required for Ku70/Ku80 association with RNA Pol II. While Ku70/Ku80 bind a number of transcription proteins (Fell and Schild-Poulter, 2015; Mo and Dynan, 2002), their dependence on AR for effective association with the transcription complex is a surprising finding of biological importance. The resulting enhancement of endogenous DNA damage suggests that already approved AR inhibitors could be used in new combination approaches for melanoma treatment. In support of this possibility are our further findings that, in the iLINCS database, similar gene expression profiles are triggered by treatment of melanoma cells with AR inhibitors and conventional DNA-damaging agents, specifically TOPO inhibitors.

The STING proinflammatory signaling cascade activated by loss of AR function can be an important determinant of tumor infiltration by immune cells (An et al., 2019; Chen et al., 2016; Woo et al., 2015). Increased cancer cell recognition and elimination by the immune system can be highly beneficial in a substantial fraction of melanoma patients (Sharma and Allison, 2015). This is consistent with bioinformatic analysis of large melanoma datasets sorted by the *AR* gene-silencing signature that we have established, as well as experimental findings with tumors formed by melanoma cells with or without AR loss. As such, AR inhibition could provide an approach to ameliorate response to immune checkpoint inhibitors, especially of “immune-excluded” and “immune desert” tumors (Chen and Mellman, 2017), in which sex hormone differences could be involved (Özdemir and Dotto, 2019).

In melanoma samples of the TCGA database, there is a 4% *AR* gene mutation frequency. However, the vast majority are missense gene mutations that do not coincide with those reported in the *AR* gene mutation database, and their functional significance will have to be assessed. Additional complexities to consider are polymorphisms of the *AR* gene (Ryan and Crespi, 2013) and the possible involvement of unrelated membrane-associated androgen sensor proteins (Thomas, 2019) that may converge with AR signaling in promoting the disease.

As suggested many years ago (Rampen and Mulder, 1980), differences in androgen levels between male and female populations are a likely determinant of their different susceptibility to the disease. However, our findings clearly indicate that melanoma cells of both male and female individuals are equally dependent on sustained AR signaling for proliferation, maintenance of genomic stability, and tumorigenesis. Sexual dimorphism in this as in other cancer types cannot be solely attributed to hormonal differences and/or their impact on individual cell types (Clocchiatti et al., 2016). At the level of individuals, the interplay between hormonal and genetic determinants of sex specification, as well as the extreme complexities of nuclear hormone receptor interactions, can result in a continuous spectrum of susceptibility to various diseases (Dotto, 2019). Irrespective of sex, our findings point to AR signaling as a significant parameter to consider for targeted approaches to melanoma management.

## Materials and methods

### Cell culture

A list of melanoma cell lines and primary melanoma cells derived from male and female patients is provided in Table S2. Early-passage (5–6) primary melanoma cell cultures (M121008, M141022, and M131206) were established from discarded melanoma tissue samples by University Research Priority Program (URPP) Live Cell Biobank (University of Zurich) with required institutional approvals. WM1366, WM983A, WM1862, and WM1552C melanoma cells were a gift from Meenhard Herlyn (The Wistar Institute, Philadelphia, PA). The YUMM1.7 melanoma cell line was provided by P-C. Ho. No further authentication of these cell lines was performed. Cell morphology and growth characteristics were monitored during the study and compared with published reports to ensure their authenticity. All melanoma cell lines and patient-derived primary melanoma cells were maintained in DMEM (Thermo Fisher Scientific) supplemented with 10% (vol/vol) FBS (Thermo Fisher Scientific) and 1% penicillin-streptomycin. Primary melanocytes were prepared from discarded human skin samples from abdominoplasty or circumcision at the departments of Plastic Reconstructive Surgery or Pediatrics, Lausanne University Hospital, with required institutional approvals (UNIL: CER-VD 222/12) and informed consent. All cells used in this study were determined to be negative for *Mycoplasma* before experiments. All cell lines were used within five passages after thawing.

### Cell manipulations and treatments

Lentiviral particle production and infections were performed as described previously (Dull et al., 1998). Details of the lentiviral shRNA vectors and sgRNA vectors used are provided in Table S6. Two different shRNAs directed against human or mouse *AR* in the pLKO.1 lentiviral vector were used to silence the gene. Melanoma cells were infected with lentiviruses for 2 h; 2 d after infection, cells were selected with 1 µg/ml of puromycin for 3 d. RNA or protein samples were collected 5 d after infection. Mouse YUMM1.7 melanoma cells used for *AR* gene silencing were previously stably infected with *RFP*-expressing lentiviral vector with blasticidin selection.

For siRNA silencing experiments, melanoma cells were transfected with *AR*- and/or *STING*-silencing siRNAs versus scrambled control siRNAs by INTERFERin (409; Polyplus Transfection) according to the manufacturer’s instructions. The details of the siRNAs used in this study are provided in Table S6.

For AR overexpression and rescue experiments, melanoma cells were stably infected with a blasticidin-resistant lentiviral vector for constitutive *AR* expression (a gift of Karl-Henning Kalland, Bergen University, Bergen, Norway) or vector control. After selection, the AR-overexpressing melanoma cells were superinfected with an *AR*-silencing or corresponding lentivirus control and selected for puromycin resistance as described above. The cell proliferation assays were performed 5 d after the second infection.

For CRISPRi downmodulation of *AR* expression, A375 melanoma cells were stably infected with a dCas9-KRAB–expressing lentivirus (pHAGE EF1a dCas9-KRAB; Kearns et al., 2014), using puromycin for selection. Cells were subsequently superinfected with lentivirus (Lenti Guide-hygro-eGFP; Ho et al., 2017) harboring scramble sgRNA or two sgRNAs targeting different regions of the *AR* promoter. Cells were analyzed 3 d after infection. The sequences of the sgRNAs are provided in Table S6.

For AR inhibitor treatment, 24 h after seeding, melanoma cells were treated with indicated concentrations of AZD3514 (Adooq Biosciences), Enzalutamide (Selleckchem), or DMSO solvent control as indicated. For DHT treatment experiments, melanoma cells were washed four times in PBS after seeding and cultured for 48 h in phenol red–free DMEM complemented with charcoal-treated FBS before treatments with DHT (MilliporeSigma) or vehicle control (EtOH) as indicated.

### Cell-based assays

Cell proliferation assays were performed by measuring the production of ATP using the CellTiter-Glo luminescent assay (Promega) as per the manufacturer’s instructions. The luminescence signals for each time point were normalized to the signal obtained at day 0.

5-Ethynyl-2′-deoxyuridine (EdU) incorporation assays were performed using Click-iT Plus EdU Imaging Kit (Thermo Fisher Scientific) following the manufacturer’s instructions. The number of EdU-positive cells was analyzed, and the data were represented as percentage of EdU-positive cells.

For clonogenicity assays, cells were plated on 60-mm dishes (1,000 cells/well; triplicate wells/condition) and cultured for 7 d. Colonies were fixed with 4% formaldehyde and stained with 1% crystal violet. The number of clones was counted using ImageJ (National Institutes of Health).

For sphere formation assays, melanoma cells were plated onto 8-well chamber slides (Corning) precoated with Matrigel (Corning). In brief, chambers were coated with 100 µl Matrigel per well and incubated for 1 h at 37°C to polymerize. 1,000 melanoma cells were plated in each well. The number of spheroids was assessed 7 d after plating through an EVOS Cell Imaging System (Thermo Fisher Scientific).

For IncuCyte cells proliferation assays, 1,000 melanoma cells per condition were seeded in triplicate into each well of a 96-well plate and allowed to attach for 12 h. The plates were mounted on IncuCyte Zoom System (Essen Bioscience), and cells were allowed to grow for the next 6 d. Images were captured at four different sectors of each well every 2 h for 6 d, and cell confluence was calculated by IncuCyte Zoom software.

For apoptosis assays, dead and proapoptotic cells were assessed using the Annexin Kit (BD Biosciences). In brief, before fixation, cells were washed with annexin-binding buffer, followed by incubation for 15 min at room temperature (RT) with annexin-Cy5 dye for staining of proapoptotic cells. After annexin incubation, cell were fixed with 4% formaldehyde and counterstained with DAPI.

Senescence-associated β-galactosidase (SA-β-Gal) activity was assessed by the use of a commercially available chromogenic assay kit (Cell Signaling) as per the manufacturer’s instructions.

### Comet assays

The extent of double-strand DNA breaks generated with or without *AR* silencing in individual melanoma cells was assessed using alkaline comet assay (single-cell electrophoresis) as described previously (Bottoni et al., 2019). Images were obtained with a Zeiss AxioImager Z1. The percentage of tail DNA per nuclei was calculated using Comet Score 1.6.1.13 software.

### IF and immunohistochemistry staining

IF staining of tissue sections and cultured cells was performed as described previously (Kim et al., 2017). Briefly, frozen tissue sections or cultured cells on glass coverslips were fixed in cold 4% paraformaldehyde for 15 min at RT. Paraffin-embedded sections were subjected to deparaffinization and antigen retrieval using a citrate-based buffer system. Samples were washed with PBS, followed by permeabilization with 0.1% Triton X-100 in PBS for 10 min and incubated with 2% BSA in PBS for 2 h at RT. Primary antibodies were diluted in fluorescence dilution buffer (2% BSA in PBS, pH 7.6) and incubated overnight at 4°C. A list of primary antibodies and dilutions used for IF is provided in Table S6. After washing three times in PBS, samples were incubated with donkey fluorescence conjugated secondary antibodies (Invitrogen) for 1 h at RT. After washing with PBS, slides were mounted with Fluoromount Mounting Medium (Sigma-Aldrich) after nuclear DAPI staining. Control staining without the primary antibodies was performed in each case to subtract background and set image acquisition parameters. IF images were acquired with a Zeiss AxioVision or Zeiss LSM880 confocal microscope with 20× or 40× oil-immersion objectives. Axiovision or Zen Black software was used for acquisition and processing of images. For fluorescence signal quantification, acquired images for each color channel were imported into ImageJ and quantified using the functions “measurement” or “particle analysis” for selection of areas or cells of interest. The fluorescence intensities are indicated as arbitrary units.

For the melanoma tissue microarray, the mean intensity of AR fluorescence in melanoma cells for each microbiopsy was measured using ImageJ. A binary image was created from the MelanA-positive cells by setting a threshold to consider only MelanA signal with pixel intensity between 36 and 255. A mask was then derived from the MelanA-positive area to mark melanoma cells, and the mean intensity of AR fluorescence was measured inside the mask. Data for melanoma tissue array data were plotted as average AR intensity of three fields per spotted tumor sample, each field comprising a group of ∼50–60 cells. Each dot represents one clinical tissue sample.

Immunohistochemical analysis was performed using a previously described protocol for prostate cancer cells (Li et al., 2018). Briefly, 4-µm-thick sections of formalin-fixed paraffin-embedded tissue blocks from different melanomas were subjected to deparaffinization using xylene, hydrated in a graded series of ethanol solutions, and subjected to antigen retrieval with 10 mM Tris/EDTA buffer solution (pH 9.0) at 100°C for 20 min. Parallel sections were permeabilized, blocked, and incubated with anti-AR antibody or anti-MelanA antibodies. Chromogenic detection was performed using a peroxidase-conjugated secondary antibody (30 min) and DAB reagents (5 min). Tissue sections were counterstained with 0.1% hematoxylin. Immunohistochemical staining was performed by an experienced laboratory of pathology in our institution. Immunohistochemical images were acquired with a Zeiss AxioVision microscope with 20× objective. Axiovision software was used for acquisition and processing of images.

### Immunoblotting and RT-qPCR

Cells were lysed in radioimmunoprecipitation assay buffer (10 mM Tris-Cl, pH 8.0, 1 mM EDTA, 1% Triton X-100, 0.1% sodium deoxycholate, 0.1% SDS, 140 mM NaCl, and 1 mM PMSF) or LDS buffer (Thermo Fisher Scientific). Equal amounts of proteins were subjected to immunoblot analysis. Membranes were sequentially probed with different antibodies as indicated in the figure legends, using an enhance chemiluminescence kit (Thermo Fisher Scientific) for detection. Details of antibodies used in this study are provided in Table S6. RT-qPCR analysis was performed as described previously (Kim et al., 2017). A list of primers used in this study is provided in Table S6.

### PLAs

PLAs were performed with a Duolink kit with provided reagents and buffers (DUO92101; Sigma-Aldrich) using the following protocol. Melanoma cells were seeded on glass coverslips in a 24-well plate. After washing with PBS three times, cells were fixed with cold 4% paraformaldehyde for 15 min at RT. The fixed cells were washed with PBS, followed by permeabilization with 0.1% Triton X-100 in PBS for 15 min at RT, incubation in blocking buffer (provided with the kit) for 2 h at 37°C in a humidified chamber, and incubation with different primary antibodies (against target proteins to be analyzed) diluted in antibody diluents overnight at 4°C. Cells were subsequently washed in buffer A three times for 15 min and incubated with the PLA probes for 1 h at 37°C in a humidified chamber. This was followed by a 10 min wash in buffer A, a ligation reaction at 37°C for 1 h, and two more washes (10 and 5 min) in buffer A. Samples were then incubated with the amplification mix for 2 h at 37°C in a darkened humidified chamber, followed by washing with 1× buffer B (10 min) and 0.01× buffer B (1 min) and processing with mounting media. Cells were counterstained with DAPI, and images were acquired with a Zeiss LSM880 confocal microscope. The number of PLA puncta (dots) per cell/nucleus was quantified using the fluorescent particle analysis with ImageJ. Details of the antibodies used in PLA assays are provided in Table S6.

### Co-IP analysis

WM1366 melanoma cells were harvested by trypsinization. Cells were resuspended in ice-cold co-IP buffer (50 mM Tris-HCl, pH 7.4, 150 mM NaCl, 1 mM EDTA, 1% NP-40, 1% Na-deoxycholate, 0.05% SDS, and protease inhibitor) and lysed by mild sonication (three 5-s pulses). The cell debris were removed by centrifugation, and the supernatant was subjected to extensive DNase I digestion. Co-IP was performed by incubating 500 µg of the lysate with 4 µg of anti-AR (5153S; CST) or anti-RNA pol II (ab26721; Abcam) antibodies in 500 µl co-IP buffer for 8 h at 4°C. A mock reaction was also performed by incubating rabbit IgG (as control) with the lysate. Prewashed Protein A magnetic beads were added, and the incubation was continued for another 1 h. The beads were washed three times with 1 ml of co-IP buffer, resuspended in SDS-PAGE sample loading buffer, and incubated for 20 min at 98°C. The samples were resolved on 8% SDS-PAGE, and immunoblotting was performed with anti-AR (06-680; Merck Millipore), anti-RNA pol II (ab26721; Abcam), and anti-Ku70 (GTX101820; GeneTex) antibodies. VeriBlot IP Detection Reagent (HRP; ab131366; Abcam) was used as secondary antibody (1:200 dilution) to selectively detect target protein bands, without interference from the denatured IgG heavy and light chains. Details of the antibodies used are provided in Table S6.

### Transcriptomic analysis

The transcriptional changes elicited in WM1366, SKMEL28, and WM115 melanoma cells with or without *AR*-silencing with two different lentiviruses versus empty vector control were assessed by Clariom D GeneChip array analysis (Thermo Fisher Scientific). 5 d after infection, RNA was extracted from the melanoma cells using Direct-zol RNA MiniPrep kit (Zymo Research) coupled with DNase treatment, and RNA quality was verified by Bioanalyzer (Agilent Technologies). 50 ng of total RNA was used as input for the preparation of single-strand cDNA using the GeneChip WT PLUS Reagent Kit (Thermo Fisher Scientific). Targets were then fragmented and labeled with the GeneChip WT Terminal Labeling Kit (Thermo Fisher Scientific) and hybridized on Human Clariom D GeneChip arrays (Thermo Fisher Scientific) at the iGE3 Genomics Platform, University of Geneva (Geneva, Switzerland). Data obtained were analyzed using TAC software (v4.0). The data generated in this study have been deposited to the public functional genomics data repository GEO, accession no. GSE138486.

GSEA for GeneChip microarray data was conducted using GSEA software with default parameters. Curated gene sets were obtained from the Molecular Signatures Database (MSigDB version 5.2, http://www.broadinstitute.org/gsea/msigdb/). A list of enriched pathway gene sets is provided in Table S4.

### Construction of the *AR*-silencing gene signature

Raw microarray expression data were preprocessed with TAC software, obtaining gene-level expression values from signal space transformation/robust multiarray average summarization. Ensemble IDs were mapped to gene symbols with Biomart. A paired differential expression analysis between control (*n* = 3) and shAR (*n* = 6) conditions was performed with Limma (default parameters), pairing together samples from each cell line. The *AR* silencing signature was constructed as the list of genes up- or down-regulated upon *AR* silencing, i.e., genes showing an adjusted P value < 0.05 and an absolute log fold-change >1 in the overall analysis as well as in each cell line separately.

### Computation of *AR*-silencing signature scores in TCGA SKCM dataset

Level 3 gene expression and clinical data for TCGA SKCM projects were downloaded from the National Institutes of Health Genomic Data Commons Data Portal (https://portal.gdc.cancer.gov). In case both primary and metastatic samples were present for the same patient, only the primary sample was retained. TPM (transcripts per million) values were transformed as log2 (TPM + 1). Scores for the sets of genes up- and down-regulated were computed with the R package GSVA v1.30.0 using default parameters. The difference between these scores was computed to obtain a unified score for the total *AR*-silencing gene signature (comprising both up- and down-regulated genes) for each patient. Each tumor was assigned a positive (+) or negative (−) score relative to the unified *AR*-silencing gene signature.

### Survival analysis

The difference between the survival of patients with a positive (+) versus negative (−) score for the *AR* silencing gene signature was tested with a log-rank test implemented in the R package survival v2.43-3. A Cox regression from the same package was used to account for the following covariates: age, sex, primary or metastatic status, and genomic subtype (*BRAF* mutant, *RAS* mutant, *NF1* mutant, or triple wild-type).

### EPIC and CIBERSORTx analyses

Cell type fractions for bulk RNA-sequencing melanoma samples from TCGA SKCM were computed with EPIC v1.1.5 using default parameters and CIBERSORTx using LM22 signature matrix and B-mode batch correction. Differences between melanomas enriched and not enriched for the *AR*-silencing gene signature were tested with Wilcoxon rank-sum tests.

### iLNCS analysis

The analysis of concordance between our in-house *AR* silencing gene signature and iLINCS chemical perturbagen signatures was performed by interrogating the iLINCS data portal (http://www.ilincs.org/ilincs/). Briefly, the iLINCS web application computes concordance as the Pearson correlation coefficient between the fold-changes of the genes in common (*n* = 21) between the query signature and the precomputed iLINCS signatures. Signatures with correlation >|0.2| and P < 0.05 are extracted and sorted by concordance. A list of top iLINCS signatures with concordance score >0.65 is shown in Table S5.

### Tumorigenesis experiments

Intradermal back injections of indicated melanoma cells were performed in 6–8-wk-old male and female NOD SCID mice (NOD.CB17-Prkdcscid/J; Jackson Laboratory). In brief, 10^6^ melanoma cells (WM1366, A375, and SKMEL28) infected with *AR* silencing versus control lentiviruses were injected (29-gauge syringe) with Matrigel (Corning; 70 µl per injection) intradermally in parallel into the left and right side of mice. Mice were sacrificed, and Matrigel nodules were retrieved for tissue analysis 16 d after injection.

For in vivo AZD3514 treatment experiments, RFP-expressing A375 melanoma cells (1 × 10^6^) were intradermally injected with Matrigel solution in the back skin of 10 male NOD SCID mice. 3 d after injection, mice were treated with either 100 µl of AZD3514 (50 mg/kg, per mouse, group of five mice) or Captisol (Ligand Technology) as vehicle control (group of four mice) for 12 consecutive days by oral gavage. The body weight of the mice was measured regularly during the treatment. Mice were sacrificed, and Matrigel nodules were retrieved for tissue analysis at the end of the treatment.

Alternatively, melanoma cells were treated with either AZD3514 (10 µM) or DMSO for 12 h in culture and injected intradermally in mice together with Matrigel as described above. The tumors were allowed to grow for 2 wk, and nodules were retrieved for tissue analysis at the end of the treatment.

Intradermal back injections of YUMM1.7-RFP cells were performed in 6- to 8-wk-old male and female mice (C57BL/6J; Jackson Laboratory). In brief, 5 × 10^5^ melanoma cells infected with *AR* silencing versus control lentiviruses were injected with Matrigel (Corning; 60 µl per injection; 29-gauge syringe) intradermally in parallel into the left and right side of mice. Mice were sacrificed, and Matrigel nodules were retrieved for flow cytometry analysis 14 d after injection. All mice were housed in the animal facility of the University of Lausanne.

### Tumor digestion, cell isolation, and flow cytometric analysis

Tumors were minced in RPMI with 2% FBS, i.v. collagenase (0.5 mg/ml; Sigma-Aldrich), and DNase (1 µg/ml; Sigma-Aldrich) and digested at 37°C for 45 min. The digested samples were then filtered through a 70-µm cell strainer and washed with FACS buffer (PBS with 2% FBS and 2 mM EDTA). The cell pellets then incubated with ACK lysis buffer (Invitrogen) to lyse red blood cells. Next, viable cells in single-cell tumor suspensions were further enriched by density gradient centrifugation (800 *g*, 30 min) at RT with 48% and 80% Percoll (GE Healthcare) and collected from the interphase of the gradient. FACS analysis was performed using an LSRII (BD Biosciences). Data were analyzed using FlowJo. The following antibodies were used for flow cytometry: anti-CD3 (17A2), anti-CD4 (RM4-5), anti-CD8α (53-6.7), anti-CD11b (M1/70), anti-CD45 (104), anti-Gr-1 (RB6-8C5), anti-FoxP3 (FJK-16s), anti-CD44 (IM7), anti-F4/80 (BM8), anti-LAG3 (C9B7W), anti-PD1 (RMP1-30), and anti-Arg1 (A1exF5). Cell populations were identified based on the following expression markers: CD4 T cells, CD45^+^ CD3^+^ CD4^+^; CD8 T cells, CD45^+^ CD3^+^ CD8^+^; regulatory T cells, CD45^+^ CD3^+^ CD4^+^ FoxP3^+^; and macrophages, CD45^+^ Gr-1^−^ F4/80^+^ CD11b^+^. Antibody details, including commercial sources, are provided in Table S6.

### Statistical analysis

Statistical testing was performed using Prism 8 (GraphPad Software). Data are presented as mean ± SEM or SD, as indicated in the legends. Statistical significance for comparing two experimental conditions was calculated by two-tailed *t* tests. For multiple comparisons of more than two conditions, one-way ANOVA was used, with Dunnett’s test to compare different test conditions to the same control. For tumorigenicity assays, wherever possible, individual animal variability was minimized by contralateral injections in the same animals of control versus experimental combinations of cells. No statistical method was used to predetermine sample size in animal experiments, and no exclusion criteria were adopted for studies and sample collection. No exclusion criteria were adopted for animal studies and sample collection. No randomization was used, and the researchers involved in the study were not blinded during sample obtainment or data analysis.

### Study approval

Melanocytes were prepared from discarded human skin samples from abdominoplasty or circumcision at the Department of Plastic Reconstructive Surgery or Pediatrics, Lausanne University, with required institutional approvals (UNIL: CER-VD 222/12) and informed consent. Benign nevi, dysplastic nevi, primary and metastatic skin sections, and melanoma tissue microarray slides were obtained from the Live Cell Biobanks of the URPP “Translational Cancer Research” (Mitchell P. Levesque, University Hospital Zurich). All samples were obtained as surplus material from consenting patients (Ek. 647/800), and the experiments were approved by the Kantonal ethics committee of Zürich (Kantonale Ethikkommission Zürich, approval no. KEK.Zh.Nr.2014-0425). No access to sensitive information was provided. All animal studies were performed according to Swiss guidelines for the use of laboratory animals, with protocols approved by the University of Lausanne animal care and use committee and the veterinary office of Canton Vaud (animal license no. 1854.4f/1854.5a).

### Online supplemental material

Fig. S1 shows double IF analysis of patient-derived melanocytic lesions; AR expression across age and between sexes in a melanoma tissue microarray; immunohistochemical analysis of AR expression in patient-derived melanocytic lesions; IF analysis of AR expression in different melanoma cell lines and primary human melanocytes with prostate cancer cells as comparison; AR protein expression in different melanoma cell lines and primary human melanocytes as detected by two different antibodies; and *AR* mRNA expression in different melanoma cell lines and primary human melanocytes. Fig. S2 shows double IF analysis of patient-derived melanocytic lesions. Fig. S3 shows silencing of *AR* in different melanoma cell lines; suppression of melanoma proliferation and self-renewal potential by *AR* silencing; and EdU incorporation assay in melanoma cells with or without *AR* silencing. Fig. S4 shows apoptosis and senescence assays in melanoma cells with or without *AR* silencing; that AR overexpression suppresses *AR* silencing effects; growth-suppressive effects of AR inhibitors on melanoma cells; and growth-stimulatory effects of DHT treatment of melanoma cells. Fig. S5 shows prevalence of stromal and immune cells in TCGA SKCM samples with and without enrichment for the *AR*-silencing gene signature; that *AR* silencing inhibits WM1366, A375, and SKMEL28 melanoma tumorigenesis; and that AZD3514 pretreatment inhibits WM1366 melanoma tumorigenesis. Table S1 is a summary of patient information of tissue microarray. Table S2 is a summary of a panel of human melanoma cell lines used in this study. Table S3 lists 155 genes up- or down-modulated by *AR* silencing in three melanoma cell lines (WM1366, SKMEL28, and WM115) from transcriptomic profiling. Table S4 lists gene sets significantly associated with differentially expressed genes in melanoma cells upon *AR* gene silencing. Table S5 lists perturbagens with concordance with *AR* signatures, including the associated P values from iLINCS database. Table S6 lists reagents and resources.

## Supplementary Material

Table S1 is a summary of patient information of tissue microarray (related to Fig. 1).

Table S2 is a summary of a panel of human melanoma cell lines used in this study (related to Fig. 1).

Table S3 lists 155 genes up- or down-modulated by AR silencing in three melanoma cell lines (WM1366, SKMEL28, and WM115) from transcriptomic profiling (related to Fig. 3).

Table S4 lists gene sets significantly associated with differentially expressed genes in melanoma cells upon AR gene silencing (related to Fig. 3).

Table S5 lists perturbagens with concordance with AR signatures, including the associated P values from iLINCS database (related to Fig. 3).

Table S6 lists reagents and resources.
